# Profiles of social-emotional competence in elementary students: A latent profile analysis

**DOI:** 10.1371/journal.pone.0350092

**Published:** 2026-06-17

**Authors:** Xiang Gao, Chang Liu, Xuyan Cui, Pukui Wang

**Affiliations:** 1 Sports Training Institute, Guangzhou Sport University, Guangzhou, Guangdong, China; 2 Guangdong Provincial Key Laboratory of Human Sports Performance Science, Guangzhou Sport University, Guangzhou, Guangdong, China; 3 Department of Physical Education and Research, Fuzhou University, Fuzhou, Fujian, China; National Institutes of Health, University of the Philippines Manila / De La Salle University, PHILIPPINES

## Abstract

**Objective:**

This study aimed to classify profiles of elementary school students based on their levels of social-emotional competence and to examine the associations of demographic variables, including gender, grade, family structure on these latent profiles.

**Methods:**

A stratified sampling method was employed to recruit 11,323 students from grades 3–6 across 42 schools in eastern, central, and western regions of China. Data were analyzed using Latent Profile Analysis (LPA) and Logistic regression. The four-profile solution was selected based on statistical indices (BIC = 1130587, Entropy = 0.912) and its interpretability.

**Results:**

(1) elementary school students’ social-emotional competence could be classified into four categories: high level group (25.8%), medium to high level group (36.2%), low to medium level group (31.3%), and low level group (6.7%). (2) With the low level group as the reference category: compared with boys, girls were more likely to be classified into the high level group (OR = 15.701, P < 0.001); relative to Grade 3 students, Grade 4 students were more likely to be classified into the high level group (OR = 17.293, P < 0.001) and into the low to medium level group (OR = 4.061, P < 0.001); non-only children were more likely than only children to be classified into the high level group (OR = 15.679, P < 0.001) and into the low to medium level group (OR = 4.074, P < 0.001); students attending urban schools were more likely than rural students to be classified into the high level group (OR = 15.576, P < 0.001) and into the medium to high level group (OR = 4.084, P < 0.001); and non-left-behind children were more likely than left-behind children to be classified into the high level group (OR = 15.671, P < 0.001) and into the medium to high level group (OR = 4.094, P < 0.001).

**Conclusion:**

Elementary school students exhibit distinct categorization patterns in social-emotional competence. These findings suggest that interventions should be tailored to address these profiles to enhance students’ social-emotional competence. It should be noted that these odds ratios are conditional on the low level group as the reference category. The low level group itself accounted for only 6.7% of the total sample, and within this already small category, the representation of certain demographic characteristics (e.g., girls, urban students) was extremely low. These two factors combined contribute to the large magnitudes of the reported odds ratios. Therefore, these values represent relative advantages compared to the low level group rather than overall population odds ratios, and should be interpreted cautiously.

## Introduction

Social-emotional competence (SEC) is defined as the integrated capacity of an individual to respond adaptively to social contexts at the cognitive, affective, and behavioral levels [1]; it is classified as a “soft skill” or “non-cognitive ability.” In recent years, with the rapid economic development and accelerated modernization in China, the complexity and volatility of society have become increasingly pronounced, placing higher demands on individual SEC. On one hand, SEC effectively promotes students’ cognitive abilities, thereby enhancing academic performance [2] and positively influencing mental health and behavioral outcomes [3,4], thus laying a solid foundation for long-term individual development. Therefore, fostering SEC in primary education holds critical significance. On the other hand, SEC is a key factor in enabling children and adolescents to adapt and thrive in complex social environments [5]. Traditional primary education systems have tended to overemphasize cognitive training while neglecting the cultivation of SEC, contributing to phenomena such as “academically focused students” or “students from small towns who excel only in test-taking.” These issues hinder holistic student development. Consequently, cultivating SEC has become a vital task in advancing quality-oriented education and deepening educational reform in China. It is also an inevitable choice for transforming primary education, with significant practical implications for improving educational quality, promoting equity, and ensuring sustainable development.

The Collaborative for Academic, Social, and Emotional Learning (CASEL) in the United States pioneered research on SEC, defining it as “the ability of individuals to recognize and manage emotions, set and achieve positive goals, appreciate others’ perspectives, build and maintain supportive relationships, make responsible decisions, and constructively address personal and interpersonal matters.” CASEL categorizes SEC into five core competencies: self-awareness, self-management, social awareness, relationship skills, and responsible decision-making [1]. The Organization for Economic Cooperation and Development (OECD), drawing on the “Big Five Personality Model” in personality psychology, defines SEC as “a subset of abilities, attributes, and characteristics related to individual success and social functioning, including beliefs about relationships with others,” and specifies six competencies: task performance, emotion management, open-mindedness, collaboration with others, and composite skills [6]. In collaboration with the United Nations Children’s Fund (UNICEF), the Department of Teacher Work of the Ministry of Education in China proposed a localized model of student SEC. Integrating the “relational being” theory from social constructivism and China’s collectivist cultural context, this model defines SEC as “the ability to recognize and manage the self, relationships with others, and relationships with collectives,” and categorizes it into six competencies: self-awareness, self-management, awareness of others, management of others, collective awareness, and collective management [7]. Collectively, these three models emphasize the multidimensional and interactive nature of SEC, with emotion regulation capacity identified as a central element bridging cognition and behavior. The present study adopts the definition of SEC advanced by the CASEL because this theoretical framework offers a clearly articulated construct and operational procedures that are suitable for large-scale screening and profile analysis in school settings. Moreover, the CASEL framework emphasizes the multidimensional interplay and contextual adaptability of SEC, thereby providing a theoretical bridge for understanding the influences of familial, school, and individual factors on children’s SEC development.

In recent years, researchers have predominantly explored the influencing factors of SEC across four dimensions: individual, family, teacher, and school. Studies indicate that emotional intelligence not only predicts psychological well-being and relationship satisfaction but also mitigates the negative impact of other risk factors on health [8], underscoring its critical role in promoting environmental adaptation and enhancing quality of life. Family environment significantly influences adolescent SEC development, with family cohesion and conflict affecting social responsibility through emotional regulation [9]. High family cohesion enhances emotional regulation, fostering social responsibility, while conflict diminishes this capacity. From the teacher perspective, educators with strong social-emotional skills organize classrooms and provide emotional and instructional support congruent with high-quality classroom climates [10]. In such safe and participatory environments, students exhibit prosocial tendencies (cooperation, helpfulness, empathy) and reduced disruptive behaviors [11]. Research on school belongingness, as noted by Eccles et al., highlights it as a key factor linked to students’ SEC, well-being, academic self-efficacy, school satisfaction, and achievement [12].

Despite widespread recognition of SEC’s importance and multidimensional explorations of its influencing factors, these studies have provided robust theoretical and practical guidance for cultivating student SEC, driving educational philosophy updates and model optimization. However, gaps persist. The multidimensional and interactive nature of SEC is often overlooked in traditional variable-centered approaches, which focus on single-dimension measurements and analyses, neglecting potential profile heterogeneity and failing to comprehensively reveal SEC’s complexity and diversity. Questions such as individuals’ overall SEC levels, characteristics of different levels, and whether influencing factors vary across groups require further investigation.

Compared to variable-centered perspectives, person-centered approaches better address these issues. Latent Profile Analysis (LPA) is a person-centered statistical technique that assigns individuals to distinct profiles according to shared response patterns, thereby capturing population heterogeneity [13]. When applied to SEC—a multidimensional construct—the technique is particularly appropriate because the dimensions that constitute SEC do not combine in a simple linear fashion. For instance, a student who scores high on social awareness yet low on relationship skills may obtain a total score comparable to that of a student whose scores are uniformly moderate, yet the two students differ markedly in underlying needs and developmental trajectories. Variable-centered approaches often overlook such compensatory or imbalanced cases; however, a growing body of evidence demonstrates that LPA effectively identifies heterogeneous groups in the domains of social emotional, behavior, and mental health [14,15]. In a seminal study of Grade 8 students, Gamboa et al.[14] uncovered four SEC profiles: Socio-emotional Adaptive (46.8%), Self-Oriented (30.3%), Other and Task-Oriented (14.5%), and Socio-emotional Non-Adaptive (8.4%). Students classified as Socio-emotional Non-Adaptive exhibited a significantly higher probability of academic failure than their counterparts (OR = 3.2). Extending this line of inquiry, Deak et al. [16] revealed that adolescent emotional traits can be grouped into highly emotional, balanced emotional, and low emotional profiles, with distinct interaction effects on mental health outcomes. Moreover, Thomas et al.[4] demonstrated that a bifactor model—comprising a global SEC factor alongside three domain-specific factors—best represents the structure of SEC, thereby affirming both its multidimensionality and its shared latent core. These findings collectively establish a theoretical foundation for hypothesizing latent profiles among primary school students.

Nevertheless, three critical gaps persist. First, the characteristics of SEC among Chinese elementary school students as revealed through latent profile analysis remain largely unexplored. Second, existing LPA investigations have predominantly focused on adolescent samples, leaving the elementary school period underexamined. Third, the mechanisms through which demographic variables (e.g., gender, grade level) shape profile distribution are poorly understood. Accordingly, the present study employs LPA to delineate latent profiles of SEC among Chinese elementary school students, elucidates the underlying profile structure, and examines how gender and grade influence profile membership. As a cross-sectional investigation, this study aims to identify the latent profiles present in the current sample rather than to trace developmental trajectories. This endeavor addresses a significant lacuna in cross-cultural research on latent classes of SEC during childhood and offers a theoretical anchor for culturally responsive and differentiated interventions.

## Research sample and methods

### Sample selection

This study employed a stratified sampling method. During the stratified sampling phase, the numbers of provinces, cities, and schools were first determined in accordance with the “4: 4: 3” ratio for Eastern: Central: Western regions, thereby aligning the sampling frame with the national distribution of elementary school students aged 6–11 reported in the China Statistical Yearbook 2022. The procedure is detailed as follows. (1)Provincial Units: taking the population of elementary school students aged 6–11 as the sampling frame, a probability-proportional-to-size (PPS) random sampling technique was employed. Specifically, three provinces (or equivalent municipalities/autonomous regions) were selected from the Eastern region, six from the Central region, and six from the Western region. (2) Municipal Units: within each selected province, all cities were stratified into two layers—“economically developed” and “economically underdeveloped”—based on per-capita GDP. Subsequently, one to two cities were randomly selected from each layer using PPS sampling, resulting in a total of 28 cities. (3) School Units: in each sampled city, primary schools were chosen under a “urban: rural = 1: 1” quota. Simple random sampling was then applied to select one to three schools per city, yielding 42 schools overall. (4) Student Units: within each selected school, intact classes were treated as the primary sampling units. Classes were randomly selected in proportion to the enrolment sizes of Grades 3–6, ensuring that the final student sample accurately reflected the grade-level distribution. A total of 12,600 questionnaires were distributed, with 11,564 returned. After excluding 241 invalid questionnaires (due to incomplete responses exceeding 20% of items, missing critical demographic variables, or patterned responses), 11,323 valid questionnaires were retained, yielding an effective rate of 98%. Data collection was conducted from 20/09/2024–28/12/2024.

### Research tool

In this study, the Social-emotional Competencies Items developed by Melnick et al.[17] were employed. This scale consists of 40 items that assess five domains: self-awareness (10 items, e.g., “Knowing when l am wrong about something” “Knowing when l can’t control something” “Knowing the emotions l feel”), self-management (14 items, e.g., “Staying calm when l feel stressed” “Setting goals for myself” “Working on assignments even when they are hard”), social awareness (5 items, e.g., “Knowing what people may be feeling by the look on their face” “Knowing how my actions impact my classmates”), relationship skills (6 items, e.g., “Sharing what l am feeling with others”“ Being welcoming to someone l don’t usually eat lunch with”), and responsible decision-making (5 items, e.g., “Thinking about what might happen before making a decision”“ Helping to make my school a better place”). It uses a Likert four-point scale, where 1 indicates “strongly disagree” and 4 indicates “strongly agree”. Higher scores denote stronger social-emotional competencies among primary school children. The original English version of the scale was translated into Chinese following established cross-cultural adaptation guidelines. Two bilingual researchers independently translated the items, and a third researcher back-translated them into English to verify conceptual equivalence. The preliminary Chinese version was then reviewed by an expert panel comprising two educational psychologists and two primary school teachers to assess cultural appropriateness and clarity. Finally, a pilot test was conducted with 120 elementary school students (Grades 3–6) to evaluate item comprehensibility, leading to minor wording refinements. In the present study, the overall Cronbach’s α coefficient for the scale was 0.947. The Cronbach’s α coefficient of the self-awareness dimension is 0.824, the self-management dimension is 0.892, the social awareness dimension is 0.760, the relationship skills dimension is 0.757, the responsible decision-making dimension is 0.761, indicating high reliability of the scale [18]. The confirmatory factor analysis fitting index is *χ*^*2*^*/df* = 4.493, RMR = 0.045, RMSEA = 0.059, IFI = 0.948, TLI = 0.931, CFI = 0.947, indicating good structural validity of the scale [19].

### Research procedures

This study employed paper-based questionnaires administered in a group setting at the class level to collect data. Prior to data collection, comprehensive communication was conducted with the sampled schools, and research protocols and ethical review applications were submitted to ensure compliance with ethical standards. Formal data collection commenced only after obtaining written informed consent from parents (or guardians) of the sampled students and securing support from the schools.

During administration, participants were provided with detailed instructions regarding the procedures and response formats. Anonymity and the sole academic purpose of the study were explicitly stated, and strict confidentiality of all data was guaranteed so as to alleviate potential concerns. Moreover, the withdrawal procedure was comprehensively explained; elementary school students were informed that they could withdraw at any point. The questionnaire included two response options—“I consent to participate” and “I do not consent.” If the latter was selected, the student was directed to quiet reading and any data collected were excluded from the analyses. Upon completion, the questionnaires were immediately collected by the administrator and securely stored to ensure both the integrity and the security of the data.

### Ethics statement

This study involves research methodology and research procedures that follow the Declaration of Helsinki. The study was approved by the Guangzhou Sports University Ethics Committee (Registration number: 2024LCLL-70). All participants agreed to participate in this research voluntarily; they provided informed consent when they completed the survey and were able to withdraw from the study freely at any time. In addition, our data were anonymized to ensure the privacy of all participants.

### Data analysis

Data were first organized and analyzed using SPSS 26.0 for reliability testing of scales, assessment of common method bias, and descriptive statistics. Confirmatory factor analysis was conducted using Amos 26.0. Latent Profile Analysis (LPA) was performed using Mplus 8.3 to identify latent profiles of social-emotional competence among elementary school students. Following the approach by Nylund et al. [20], model parameters were estimated starting from a two-class baseline model, incrementally increasing the number of classes. Model fit was evaluated using indices such as the Akaike Information Criterion (AIC), Bayesian Information Criterion (BIC), and Adjusted Bayesian Information Criterion (aBIC), where lower values indicate better fit. The Entropy index, reflecting classification precision, was considered acceptable when ≥0.8 (indicating ≥90% accuracy) [21]. The Lo-Mendell-Rubin likelihood ratio test (LMR) and Bootstrap likelihood ratio test (BLRT) were used to compare models, with p-values <0.05 indicating superior fit for the k-class model over the k-1 class model [22]. To account for the nested structure of the data (students clustered within schools), all LPA models and subsequent analyses were estimated using the TYPE = COMPLEX option in Mplus, with school ID specified as the cluster variable. This approach computes robust standard errors that correct for the non-independence of observations Subsequent analyses examined the relationship between derived latent profiles and predictor variables using the R3STEP method in LPA, focusing on associations with demographic variables such as gender and grade, only child status, school location, and left-behind status.

## Results

### Common method bias test

This study employed Harman’s single-factor test to assess common method bias. All measured variables were included in an exploratory factor analysis without rotation to extract the variance explained by unrotated factors. Results indicated that five factors had eigenvalues greater than 1. The variance explained by the first factor was 32.996%, below the 40% threshold [23], suggesting that common method bias was not a serious concern.

### Descriptive statistics analysis

The statistical results in Table 1 show that the total score of SEC in elementary school students is significantly and positively correlated with self-awareness, self-management, social awareness, relationship skills, and responsible decision-making.

**Table 1:** Correlation analysis of social-emotional competence (N = 11323).

Variables	M	SD	1	2	3	4	5
1. SCE	121.00	22.34	1.00				
2. Self-awareness	30.01	6.21	0.865^**^	1.00			
3. Self-management	42.03	8.80	0.921^**^	0.714^**^	1.00		
4. Social awareness	14.92	3.50	0.821^**^	0.657^**^	0.691^**^	1.00	
5.Relationship skills	18.25	3.98	0.818^**^	0.617^**^	0.663^**^	0.640^**^	1.00
6. Responsible decision-making	15.78	3.43	0.816^**^	0.615^**^	0.683^**^	0.636^**^	0.711^**^

Note: ^**^P ＜ 0.01.

### Latent profile analysis

Starting with a one-category baseline model, we incrementally added one latent category at a time, ultimately forming five distinct category models. As the number of latent categories increased, the values of AIC, BIC, and aBIC gradually decreased. However, the smallest category probability in the five-category model was 4.5%. Although a five-class solution was statistically identifiable, this small class size (4.5%) raised concerns about the stability of parameter estimates and the interpretability of the class [24]. Further inspection revealed that the five-class solution essentially split one of the classes from the four-class solution into two smaller profiles that did not demonstrate conceptually distinct patterns across the five SEC dimensions. Considering both statistical indices and substantive interpretability, the four-class model was retained as the optimal solution. For model fit results, see Table 2.

**Table 2:** Comparison of indicators across category models.

Model	AIC	BIC	aBIC	Entropy	LMR (*p*)	BLRT (*p*)	Category Probabilities
1	1262801	1263388	1263133				
2	1164347	1165235	1164850	0.939	<0.05	<0.05	51.1%/48.9%
3	1138657	1139845	1139330	0.918	<0.05	<0.05	26.6%/43.4%/30.0%
4	1129098	1130587	1129942	0.912	<0.05	<0.05	6.7%/ 31.3%/36.2%/25.8%
5	1125091	1126881	1126105	0.875	<0.05	<0.05	4.5%/22.6%/28.9%/24.9%/19.1%

**Fig 1:**
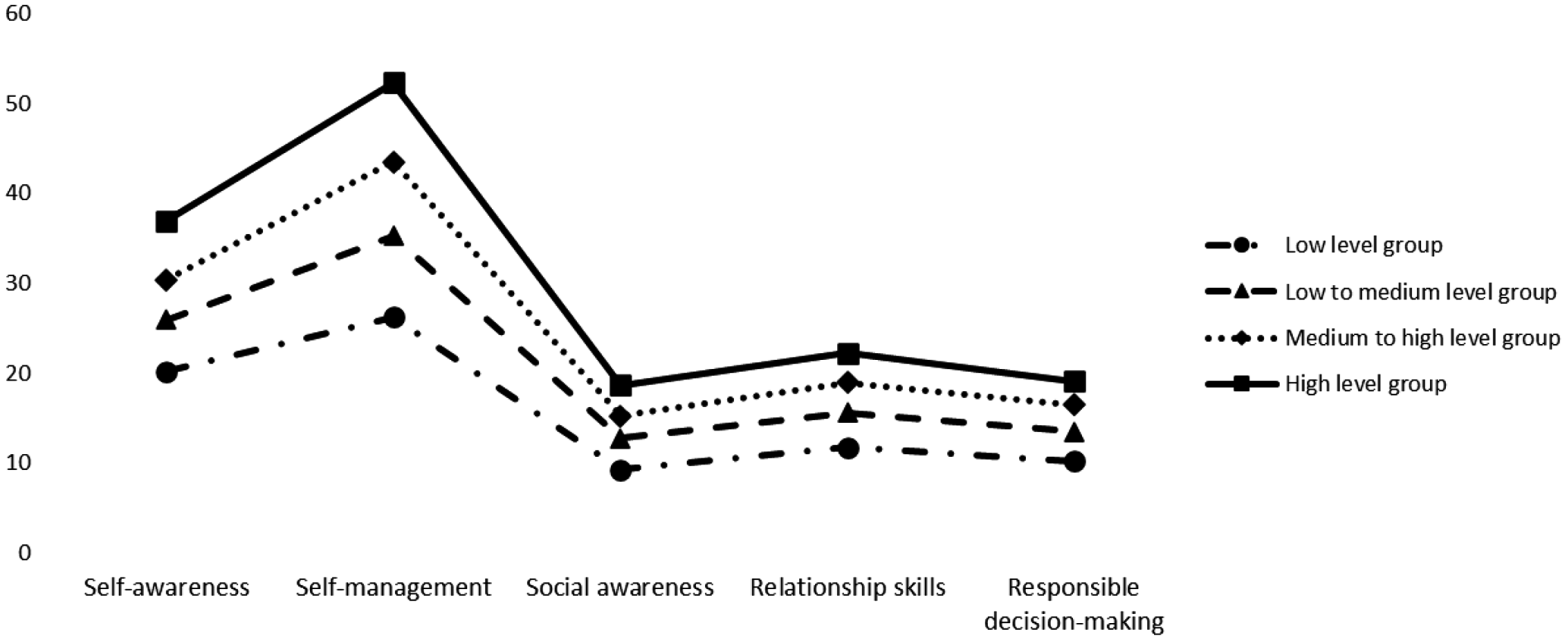
Differences in dimension scores across latent profiles of SEC.

One-way ANOVA was conducted to examine differences in the dimensions of social-emotional competence across the four latent profiles, with results presented in Table 3. Significant differences were found in self-awareness scores across the four profiles (F = 241.92, η^2^ = 0.021, P ＜ 0.001). Follow-up simple effects analysis revealed that: Profile 2 had significantly higher self-awareness scores than Profile 1. Profile 3 had significantly higher self-awareness scores than Profiles 2 and 1. Profile 4 had significantly higher self-awareness scores than Profiles 3, 2, and 1. A similar pattern was observed for self-management, social awareness, relationship skills, and responsible decision-making. Based on these results, the profiles were labeled as follows: Profile 1 was labeled as the low level group, Profile 2 was labeled as the low to medium level group, Profile 3 was labeled as the medium to high level group, Profile 4 was labeled as the high level group. See Fig 1 for differences in dimension scores across latent profiles.

**Table 3:** Differences in dimensions of social-emotional competence across latent profiles among elementary students.

	Class 1	Class 2	Class 3	Class 4	η^2^	F
Self-awareness	2.01 ± 0.13	2.59 ± 0.16^a^	3.04 ± 0.18^ab^	3.70 ± 0.08^abc^	0.021	241.92^***^
Self-management	1.87 ± 0.11	2.52 ± 0.19^a^	3.11 ± 0.25^ab^	3.74 ± 0.11^abc^	0.031	291.91^***^
Social awareness	1.85 ± 0.08	2.55 ± 0.14^a^	3.04 ± 0.16^ab^	3.73 ± 0.08^abc^	0.014	220.53^***^
Relationship skills	1.96 ± 0.17	2.60 ± 0.22^a^	3.16 ± 0.28^ab^	3.70 ± 0.15^abc^	0.045	74.18^***^
Responsible decision-making	2.03 ± 0.10	2.70 ± 0.10^a^	3.30 ± 0.11^ab^	3.81 ± 0.03^abc^	0.008	361.24^***^

Note: Compared with Class 1, there was a significant difference (P < 0.05), marked as a; Compared with Class 2, marked as b. Compared with Class 3, marked as c. ^***^, P < 0.001.

### Demographic characteristics of latent profiles of SEC in elementary students

To investigate the associations of demographic variables with social-emotional competence (SEC), multinomial logistic regression was conducted. The latent profiles of SEC were entered as the outcome variable, with the low level group serving as the reference category. Predictor variables included gender (Boy = 0, Girl = 1), left-behind status (Left-behind = 0, Non-left-behind = 1), school location (Rural = 0, Urban = 1), only child status (Only child = 0, Non-only child = 1), and grade level (treated as a categorical variable with Grade 3 as the reference category, and dummy variables created for Grades 4, 5, and 6). To account for the nested structure of the data (students clustered within schools), robust standard errors were estimated using the clustering option in Mplus. Odds ratios (OR) were calculated to assess the associations of these demographic variables with SEC profiles.

As shown in Table 4, girls were more likely than boys to be classified into the low to medium level group, medium to high level group, high level group. Non-only children exhibited higher SEC development than only children. Students from urban schools were more likely than those from rural schools to be in the low to medium level group, medium to high level group, high level group. Non-left-behind students demonstrated higher SEC development than left-behind students. Additionally, older students (Grades 4–6) were more likely than younger students (Grade 3) to be in the low to medium level group, medium to high level group, high level group.

**Table 4:** Multinomial logistic regression analysis of demographic variables on four latent profiles of SEC.

Predictor Variables	C1 vs. C2		C1 vs. C3		C1 vs. C4	
	b(SE)	OR	b(SE)	OR	b(SE)	OR
Gender						
Boy = 0	1.413^#^ (0.051)	4.109	0.701^#^ (0.048)	2.016	2.754^#^ (0.083)	15.701
Grade (Third = 0)						
Fourth	1.401^#^ (0.064)	4.061	0.733^#^ (0.059)	2.082	2.850^#^ (0.090)	17.293
Fifth	0.940^#^ (0.053)	2.559	0.442^#^ (0.046)	1.556	1.629^#^ (0.073)	5.099
Sixth	1.133^#^ (0.056)	3.104	0.555^#^ (0.050)	1.742	2.021^#^ (0.083)	7.547
Only child						
Yes = 0	1.405^#^ (0.051)	4.074	0.696^#^ (0.048)	2.005	2.752^#^ (0.083)	15.679
School location						
Rural = 0	0.696^#^ (0.048)	2.007	1.407^#^ (0.051)	4.084	2.746^#^ (0.082)	15.576
Left-behind children						
Yes = 0	0.695^#^ (0.048)	2.004	1.410^#^ (0.051)	4.094	2.752^#^ (0.083)	15.671

Note: C1: Low level group, C2: Low to medium level group, C3: Medium to high level group, C4: High level group; OR=odd ratios.

# *P* ＜ 0.001.

## Conclusion

### Latent profiles of SEC

Using a person-centered approach, this study applied latent profile analysis to explore the latent profiles of SEC among elementary school students. The results revealed four distinct profiles of SEC: low level group, low to medium level group, medium to high level group, high level group. The medium to high level group was the most prevalent, comprising 36.2% of the sample, followed by the low to medium level group (31.3%), high level group (25.8%), and low level group (6.7%). This distribution suggests that overall SEC development among Chinese elementary school students is relatively high.

Further analysis of group characteristics indicated that students in the low level group exhibited significant deficiencies across all five dimensions of SEC (self-awareness, self-management, social awareness, relationship skills, and responsible decision-making). These deficiencies may be associated with factors such as family environment, limited school resources, and personal experiences. Students in the low to medium level group showed certain strengths in some dimensions but had considerable room for improvement in others, indicating uneven SEC development. Those in the medium to high level group performed well in most dimensions but still had potential for further growth in specific areas. Students in the high level group demonstrated exceptional performance across all dimensions, reflecting strong SEC.

It is noteworthy that the four profiles identified in this study are characterized primarily by different levels of overall SEC rather than by qualitatively distinct patterns across the five dimensions (e.g., high in social awareness but low in self-management). This pattern is attributable to the moderate to high positive correlations observed among the five SEC dimensions (see Table 1), which tend to produce “level” classes in latent profile analysis when indicators are substantially correlated. While this may limit the identification of more heterogeneous “shape” profiles, the resulting classification along a severity continuum holds significant practical value. It provides a clear and interpretable framework for screening and tiered intervention: educators can readily identify students falling into different risk or strength levels and allocate resources accordingly. Students in the low level group may require intensive, multi-systemic support, whereas those in the medium to high and high level groups may benefit from universal enrichment or targeted strength-building programs. Future research with more heterogeneous samples or additional indicators capturing distinct facets of SEC might reveal more qualitatively differentiated profiles.

### Associations of demographic variables with SEC profiles in elementary students

This study found that elementary school girls exhibit significantly higher social-emotional competence (SEC) than boys, consistent with prior research [14,25]. The factors that may explain this gender difference can be systematically explored from four dimensions. Firstly, relevant research shows that girls demonstrate higher social skills and emotional regulation abilities as early as the preschool years. Their stronger emotional expression and understanding in peer interactions lay a foundation for social interactions during the school-age years [26]. Further analysis indicates that girls’ self-awareness and self-management abilities are significantly and positively correlated with academic performance, suggesting that SEC is associated with academic achievement [26]. Secondly, gender differences are reinforced in the process of family socialization. Research has shown that parental educational strategies differ significantly between boys and girls. For instance, parents are more likely to emphasize competitive behaviors in interactions with boys, while providing greater emotional support and empathy training to girls [27]. This differential parenting may be related to girls’ advantages in emotional communication and social interaction. Third, The advantage of girls in SEC may also be related to their psychological development during adolescence. Girls are more susceptible to emotional and social influences during adolescence, which may enhance their abilities in emotional regulation and social interaction [28]. Additionally, girls often exhibit higher emotional sensitivity and adaptability when facing stress and challenges, potentially contributing to their superior SEC compared to boys [29]. Finally, cultural expectations may also reinforce gender differences in SEC. In some cultural contexts, girls are expected to exhibit higher SEC, which can further amplify these differences [30]. For example, research in China shows that girls exhibit a stronger negative correlation between social dominance orientation and prosocial behavior compared to boys [30]. This cultural norm, through implicit educational mechanisms, reinforces gender role expectations in emotional expression and social interaction. In summary, girls’ advantages in SEC development are the result of multiple factors, including socialization processes, educational strategies, psychological development characteristics, and cultural and social environments. These combined influences enable girls to excel in SEC.

The present study reveals significant grade differences in the SEC of elementary school students. This finding aligns with existing supportive evidence on Social-emotional learning(SEL) reported in the literature [1]. SEL programs are widely used in early education to enhance children’s social skills, emotional regulation, and self-regulation. These programs not only positively influence SEC in the short term but also improve academic performance and behavior in the long term [31]. Students in higher grades exhibited superior performance in the dimensions of self-management, social awareness, and relationship skills. This phenomenon may be related to their increased exposure, during the learning process, to instructional activities that require teamwork and emotional exchange, such as group discussions and project-based collaboration. These activities may be associated with enhanced students’ competencies in social awareness, relationship skills, and responsible decision-making. In recent years, SEL programs have been widely promoted globally, including in China. Through systematic curriculum design and teaching practices, these programs may be associated with improved SEC. They may not only assist students in better managing their emotions but also support development in social awareness, relationship skills, and responsible decision-making. Additionally, SEL programs have been shown to be associated with enhanced peer relationships and students’ social-emotional skills. A study evaluating a school-based SEL program for children aged 9–12 found that it significantly improved students’ social skills, peer connections, and well-being [32]. This indicates that school-based SEL programs may effectively support peer relationships during school transitions. As students’ progress in grade level, their social circles in school expand, and peer relationships increasingly may be associated with SEC. Older students are more likely to learn how to handle interpersonal relationships, resolve conflicts, and express emotions through peer interactions, which may enhance their SEC across all dimensions. Peer relationships also provide emotional support and a sense of belonging, aiding students in better adapting to school life and social environments. In special education settings, peer relationships are significantly associated with student motivation. Research shows that peer relationships may be associated with learning motivation through mechanisms such as relatedness, cooperation, and participation [33]. This further underscores the importance of peer relationships in students’ academic and social-emotional development.

This study found that elementary school students’ SEC varies significantly by school location. Compared to students in the high level group, rural school students were more likely to be in the low level group, low to medium level group, medium to high level group than urban school students. This aligns with prior research indicating that urban students exhibit higher SEC development than rural students. Urban students’ superior SEC development is supported by multiple studies. Urban students benefit from stronger social support systems, which may be associated with positive SEC development [34]. Urban schools typically offer richer educational resources, better teaching facilities, and more extracurricular activities, providing students with ample opportunities to practice social awareness and relationship skills, thereby fostering a favorable environment for SEC development [35]. In contrast, rural students face more challenges in SEC development. Research shows that rural students, often from economically disadvantaged families, lack sufficient social support and educational resources, which may negatively affect their SEC development [36]. Additionally, rural students receive less teacher feedback, further limiting their SEC improvement [37]. Urban families and schools place greater emphasis on holistic student development, including SEC cultivation. In contrast, rural educational philosophies may prioritize academic performance over SEC. Notably, rural students’ SEC can improve with enhanced social support systems and teacher training in rural schools [38]. Increasing rural students’ participation in extracurricular activities may also be associated with higher SEC [39]. In summary, disparities in SEC development between urban and rural students may stem from unequal resource distribution and social support systems. Policy interventions and resource investment may mitigate these gaps and promote comprehensive student development [40].

Research findings indicate that elementary school students who are not only children are more likely to be in the low to medium level group, medium to high level group, and high level groups than those in the low-competence group. Non-only children benefit from the companionship of siblings, which provides more opportunities for social interaction, potentially fostering stronger social skills and emotional understanding in early development [41]. Moreover, non-only children may experience more conflict and cooperation within the family, which enhances their emotional regulation and problem-solving abilities [42]. Parents’ parenting styles and emotional support play a crucial role in children’s emotional regulation and social adaptation, and non-only children, with siblings, may more easily access diverse emotional support [42]. Notably, the emotional and behavioral performance of non-only children may also relate to their roles and responsibilities within the family. Research shows that non-only children often take on more responsibilities, which may help develop stronger self-regulation and social responsibility [43]. In school settings, non-only children may exhibit better SEC. They are more likely to establish good peer relationships, potentially contributing to more mature emotional and behavioral performance [43]. However, some studies suggest that only children may have higher SEC than non-only children. Family environment significantly is associated with children’s social and emotional development. Only children typically enjoy more family resources and parental attention, which may benefit their SEC development [44]. Additionally, only children’s SEC development may be influenced by parenting styles. Emotional support and involvement from parents positively are associated with children’s social and emotional development, and only children usually receive more parental support and involvement [45]. This support may be associated with better understanding and management of their emotions, thereby potentially relating to enhanced SEC. Family capital theory categorizes family capital into economic, cultural, and social capital. Only-child families often have more economic resources, while non-only-child families may have advantages in cultural and social capital. Individuals’ needs for these three types of capital vary across different developmental stages, which may be related to the discrepancies in SEC development between only and non-only children. Future research should further explore the interplay of these factors to comprehensively understand the differences in SEC development between only and non-only children.

Left-behind children are defined as minors under the age of eighteen whose mother or father, or both parents, have been absent from home for more than six consecutive months owing to migrant work, and whose daily guardianship is assumed by grandparents, other relatives, or non-parental caregivers [46]. Research findings indicate that non-left-behind elementary school students exhibit significantly higher SEC than left-behind students, consistent with prior studies [46]. This phenomenon is particularly pronounced in rural areas of China. Based on Bronfenbrenner’s ecological systems theory, left-behind children face adaptation challenges at multiple levels, including family, school, and society. Studies show that the long-term separation from parents and the lack of direct parental care and support may lead to emotional support deficits, which may negatively impact their self-identity and self-esteem [47]. This lack of emotional support places left-behind children at a disadvantage in SEC development. These children often exhibit higher levels of anxiety and depression, which are associated with family functioning and self-esteem [48]. Further research highlights that left-behind children also experience mental health issues, including emotional disorders [49]. These mental health problems are closely associated with parents’ prolonged absence, particularly when both parents are working away from home, exacerbating the severity of mental health issues [50]. Additionally, left-behind children are more prone to emotional and behavioral problems, especially in impoverished rural areas. Studies find that the prevalence of emotional and behavioral problems among left-behind children is significantly higher than among their non-left-behind peers, largely attributed to caregivers’ parenting styles and family environment [51]. Left-behind children’s social adaptation abilities are also affected. Research indicates that they have lower school adaptation abilities and more strained relationships with peers [52]. They often lack a sense of school belonging and may have distant relationships with teachers. Studies show that school belongingness and teacher-student relationships significantly are associated with students’ SEC development, and left-behind children’s disadvantages in these areas may negatively associated with their SEC development. These issues not only affect their mental health but also have a negative impact on their social functioning [53]. Family economic capital is one of the important factors associated with students’ SEC. Left-behind children’s families typically have relatively poor economic conditions, making it difficult to provide abundant learning resources and emotional support, which may be associated with limited potential for SEC development. The division of labor where one parent works away while the other stays home to accompany the children may be associated with acquired family economic capital but may be related to weakened family interaction and communication, being associated with reduced direct education and emotional support for children, and also may be associated with negative impacts on students’ SEC development.

### Practical implications

The identification of four distinct SEC profiles—low, low-to-medium, medium-to-high, and high—offers a practical framework for tiered intervention in school settings. For students in the low level group (6.7%), who exhibit deficits across all five dimensions, intensive, multi-systemic support is warranted. Schools should consider coordinating with families and community mental health services to provide targeted skill-building in self-awareness and self-management, as these foundational domains may be the most actionable entry points. For the low-to-medium level group (31.3%), characterized by uneven development, compensatory interventions focusing on specific weaker dimensions—particularly social awareness and relationship skills—could be delivered through small-group workshops or structured peer activities. Students in the medium-to-high level group (36.2%) demonstrate generally adequate SEC but may benefit from universal enrichment programs aimed at consolidating strengths and preventing regression. The high level group (25.8%) could be engaged as peer mentors or student leaders, providing them with opportunities to further refine their skills through teaching others.

The profile proportions also suggest potential screening thresholds. For instance, the 6.7% prevalence of the low level group could serve as a benchmark for identifying students in need of intensive support, while the combined 38.0% of students in the low and low-to-medium groups may represent a target population for universal or targeted prevention efforts. Among the five SEC dimensions, self-awareness and self-management emerged as particularly critical, as they showed the clearest differentiation between adjacent profiles and are often prerequisites for developing social awareness and relationship skills. Educational interventions may therefore prioritize these two domains as foundational targets.

### Conclusion

(1) This study identified four latent profiles of social-emotional competence (SEC) among elementary school students: high level group, medium to high level group, low to medium level group, and low level group. The medium to high level group was the most prevalent, while the low level group was the least prevalent. Significant differences were found across all five dimensions of SEC among these groups.
(2) Demographic variables such as gender, grade level, school location, only-child status, and left-behind status were significantly associated with students’ SEC profiles.

### Research limitations and future directions

(1) The sample of this study was confined to elementary school students, and it did not encompass a broader age range or students from other educational stages. This homogeneity in sampling may restrict the generalizability of the findings, particularly when the conclusions are extended to other age groups or educational levels. Future investigations should expand the sample to include students of different age brackets and should conduct in-depth analyses of discrepancies in SEC development among diverse groups, thereby further enhancing the external validity of the results.
(2) SEC is a multidimensional and integrated competence. The present study primarily examined the independent effects of single variables—namely, gender, grade, and family structure—on SEC, yet it did not fully explore possible interaction effects and underlying mechanisms among these core variables (e.g., gender and family structure, urban–rural differences and grade). Moreover, the scope of potential predictors was limited; important environmental factors such as pedagogical approaches employed by teachers, the instructional climate of schools, and community resources were not incorporated into the analytical framework. Future research is required to construct multilevel models that specifically investigate the complex interactive effects of key predictors on developmental pathways of SEC and to examine a wider array of environmental variables, with the aim of comprehensively elucidating the complexity of SEC development.
(3) Owing to the cross-sectional design of this study, the developmental trajectory of SEC among elementary school students could not be tracked dynamically. For instance, observed differences in SEC levels across grades merely reflect inter-grade comparisons and do not constitute longitudinal developmental evidence. Future studies are encouraged to adopt longitudinal designs to trace the developmental process of SEC in elementary school students dynamically, to analyze its developmental trajectory, and to examine its relationships with variables such as academic achievement and mental health. Longitudinal data will facilitate the disclosure of the intrinsic mechanisms underlying SEC development and its key determinants.
