# Supplementary material for: Profiles of social-emotional competence in elementary students: A latent profile analysis
Source: PLoS One. 2026 Jun 17;21(6):e0350092. doi: 10.1371/journal.pone.0350092 (PMC13274852; doi:10.1371/journal.pone.0350092)
Supplement: Attachment — Submitted filename: Response to Reviewers.pdf [file pone.0350092.s005.pdf]

Replies to the Reviewer

Thank you very much for your comments on this paper. Your comments have important guidance for the improvement of this paper and our scientific research work. These pertinent comments are a great encouragement and support to us. After careful consideration and discussion, we make the following answers according to the review comments. We hope our replies and revision to the manuscript would satisfy the requirements from you. Please feel free to let me know if you have further questions on our revised manuscript. Thank you very much for your consideration and best wishes to you.

We look forward to hearing from you at your earliest convenience.

Question 1: Please ensure that the terminology is consistent throughout the manuscript. The response indicates that "subtypes" was changed to "profiles," but some instances of "subtype" still appear in the cover letter and manuscript. Please replace these with "profile" or "profile membership" as appropriate.

Response: Thank you very much for your thorough review and valuable comments on our manuscript. Regarding the terminology inconsistency you pointed out, we have conducted a comprehensive check of the entire manuscript and the cover letter. All remaining instances of "subtype" and "subtypes" have been uniformly revised to "profile" or "profile membership" according to the context, ensuring consistency with the title and the main text. The terminology is now consistent throughout the manuscript.

Question 2: Please recheck the odds ratios in the abstract and Table 4 against the original Mplus output. Several OR values remain very close to one another, such as 2.754, 2.752, 2.746, and 2.752. If these are correct, the response to the reviewer should explicitly state that the values were verified against the original output. If not, please correct them accordingly.

Response: Thank you very much for your continued attention and rigorous correction. Regarding your request to re-check the odds ratios (ORs) in the abstract and Table 4 against the original Mplus output, we have performed another careful verification. After comparing each OR with the original analysis output, the reported ORs in the abstract and Table 4 (including 2.754, 2.752, 2.746, 2.752, etc.) are all accurate and fully match the original results. Although these values are statistically close, they

reflect the true model estimates. This situation has been explicitly clarified in our response.

Question 3: Please correct the typographical issue in Table 3. The Social awareness row appears to show "3.04 ± 016" and should likely be "3.04 ± 0.16."
Response: Thank you very much for pointing out the typographical error in Table 3, which has been very helpful for improving the quality of our manuscript. We have corrected the "Social awareness" row in Table 3 from the previously incorrectly written "3.04 ± 016" to "3.04 ± 0.16". This error was due to our negligence in formatting, and we greatly appreciate your careful correction.

Question 4: Please soften any remaining causal language, since the study is cross sectional. Phrases such as "impact of demographic variables," "effects of these demographic variables," and "significantly influenced SEC" may be revised to "were associated with SEC profiles," "were linked to profile membership," or "students with these characteristics were more likely to belong to specific profiles."
Response: Thank you for pointing this out. Following your suggestion, we have revised all causal expressions (e.g., "impact," "effects," "significantly influenced") to associative phrasing. In addition, for the causal wording originally present in the cited references, we have retained them as citations out of respect for previous research findings and because they do not represent the conclusions of the present study, ensuring that no misleading causal implications arise from the overall context.

Question 5: Please standardize the Data Availability Statement across the manuscript, cover letter, and submission form. Since the data involve minors, the statement should clearly identify the ethics committee, the valid contact information, the criteria for data access, and the requirement for a confidentiality or data use agreement. Please also check the email formatting in the cover letter, as there appears to be an extra space in the ethics committee email address.
Response: Thank you very much for your detailed review of our manuscript. Your comments have been highly instructive for improving the manuscript. We have standardized the Data Availability Statement across the manuscript, cover letter, and submission form to ensure consistent wording. Moreover, we have clearly specified in the statement the full name of the ethics committee, a valid contact email address, the specific conditions for data access, and the requirement to sign a data confidentiality

agreement, in order to comply with research norms involving minor data. In addition, we have removed the extra space in the ethics committee email address in the cover letter to ensure accurate contact information. Once again, thank you for your valuable feedback. We believe the above revisions have further enhanced the transparency and standardization of the manuscript.

Question 6: In the Data Analysis section, the sentence referring to demographic variables "such as gender and age" should be revised because age was not the actual predictor reported. Please list the actual variables used, including gender, grade, only child status, school location, and left behind status.

Response: Thank you very much for your careful review of our manuscript and for identifying the inaccuracy in the listing of demographic variables in the Data Analysis section. In response to your comment, we have revised the "Data Analysis" section, replacing the inaccurate phrase "such as gender and age" with the actual demographic variables included in the analysis, namely gender, grade, only child status, school location, and left-behind status, thereby ensuring full consistency with the variables reported in the Results section.
