# Supplementary material for: Profiles of social-emotional competence in elementary students: A latent profile analysis
Source: PLoS One. 2026 Jun 17;21(6):e0350092. doi: 10.1371/journal.pone.0350092 (PMC13274852; doi:10.1371/journal.pone.0350092)
Supplement: Attachment — Submitted filename: Response to Reviewers.docx [file pone.0350092.s002.docx]

**Replies to the Reviewer**

Thank you very much for your comments on this paper. Your comments have important guidance for the improvement of this paper and our scientific research work. These pertinent comments are a great encouragement and support to us. After careful consideration and discussion, we make the following answers according to the review comments. We hope our replies and revision to the manuscript would satisfy the requirements from you. Please feel free to let me know if you have further questions on our revised manuscript. Thank you very much for your consideration and best wishes to you.

We look forward to hearing from you at your earliest convenience.

Question 1: Terminology is still inconsistent, as “profiles” is used in the title while “subtypes” remains in key parts of the manuscript.

Response: Thank you very much for your thorough review of our manuscript. We conducted a comprehensive review of the entire manuscript and uniformly revised all instances of “subtypes” to “profiles” to ensure consistency with the title. Terminology across the manuscript is now unified.

Question 2: Table 3 still contains a visible formatting or typographical artifact that should be corrected.

Response: Thank you for pointing this out. We carefully reviewed and revised Table 3 to ensure that the content is complete, the formatting is standardized, and the presentation meets journal requirements. In addition, we rechecked all other tables in the manuscript and confirmed that they are correct.

Question 3: although the Discussion has been improved, the language should undergo one final review to ensure that no causal implications are made from cross-sectional findings.

Response: Thank you very much for your detailed and rigorous review of this manuscript. We conducted a sentence-by-sentence review of the Discussion section and revised any expressions that could imply causal inference to phrasing that describes associations (e.g., “may be associated with,” “may be related to”), ensuring that the language aligns with the cross-sectional study design. For those causal expressions that could not be modified because they originated from cited references, we retained them in the original text in the form of citations, while ensuring that the overall context does not create misleading causal implications.
