# Supplementary material for: Profiles of social-emotional competence in elementary students: A latent profile analysis
Source: PLoS One. 2026 Jun 17;21(6):e0350092. doi: 10.1371/journal.pone.0350092 (PMC13274852; doi:10.1371/journal.pone.0350092)
Supplement: Attachment — Submitted filename: Response_to_Reviewers_auresp_2.docx [file pone.0350092.s003.docx]

**Replies to the Reviewer 3**

Thank you very much for your comments on this paper. Your comments have important guidance for the improvement of this paper and our scientific research work. These pertinent comments are a great encouragement and support to us. After careful consideration and discussion, we make the following answers according to the review comments. We hope our replies and revision to the manuscript would satisfy the requirements from you. Please feel free to let me know if you have further questions on our revised manuscript. Thank you very much for your consideration and best wishes to you.

We look forward to hearing from you at your earliest convenience.

Question 1: Consider “Profiles” rather than “Subtypes” if you want alignment with common LPA language

Response: We sincerely appreciate your pointing this out. We have revised the wording in the title accordingly.

Question 2 : The abstract appears to repeat identical odds ratios (e.g., OR = 2.75 and OR = 1.41) across multiple predictors (gender, only-child status, urban/rural, left-behind). That pattern is statistically unlikely and reads like a copy/paste or transcription error.

Response: Thank you very much for identifying this critical error. An important contributing factor was that we initially retained two decimal places for all values when presenting the data, and some figures happened to be similar; however, this approach indeed created potential for misinterpretation. In the revised manuscript, we have corrected each odds ratio value in the abstract one by one and uniformly retained three decimal places to ensure the accuracy and clarity of the data.

Question 3: Consider adding 1–2 fit statistics (e.g., entropy, rationale for choosing 4-class solution) in a very compact way.

Response: We fully accept your suggestion. We have concisely added the key fit indices for selecting the four-profile solution, namely the Bayesian Information Criterion (BIC) and Entropy, at the end of the Methods section in the abstract. This revision aims to enable readers to grasp the statistical basis for model selection more quickly, reflecting the rigor of the study while maintaining the conciseness of the abstract.

Question 4: you sometimes imply “developmental profiles,” but the design is cross-sectional. I’d recommend consistently saying “latent profiles in this sample” rather than developmental trajectories (unless you add longitudinal data).

Response: Thank you very much for your thorough and detailed review of this manuscript. We fully accept and have adopted your suggestion. We conducted a comprehensive check of the wording in the Introduction section and uniformly revised all expressions that could potentially imply development or trajectory (e.g., "developmental profiles," "developmental characteristics") to more precise terms consistent with the cross-sectional study design (e.g., "latent profiles," "characteristics"). Furthermore, we added a sentence clarifying the nature of the research design at the corresponding position near the end of the Introduction, explicitly stating that this study is a cross-sectional investigation aimed at identifying the latent profile structure in the current sample rather than tracing developmental trajectories. This modification aligns the terminology throughout the manuscript closely with the research method, helping readers accurately understand the nature and contribution of this study.

Question 5: It would help to clarify the unit of inference: students are nested in classes/schools—did you account for clustering anywhere (e.g., complex survey design in Mplus, robust SEs)?

Response: We sincerely appreciate you pointing this out. We have confirmed that the data analysis employed the TYPE = COMPLEX command in Mplus, with school as the cluster variable to correct the standard errors. We have supplemented the "Data Analysis" section with a relevant explanation, clarifying this procedure to ensure the robustness of the parameter estimates.

Question 6: I’d like one extra sentence on translation/adaptation (if applicable): Was the instrument administered in Chinese? Was there back-translation or pilot testing?

Response: We fully accept your suggestion. We have added a detailed description of the scale translation and adaptation process in the "Research Tool" section, including independent translation by two researchers, back-translation, expert review, and a small-sample pilot test. This addition reflects the standardization of cross-cultural research and the cultural adaptability of the instrument.

Question 7 : There is an internal inconsistency in the rationale for restrictions

Response: We sincerely appreciate you pointing this out. We have consolidated the data availability statement into a unified expression, clarifying that the data are not publicly available due to the protection of minors' privacy as required by the ethics committee. The statement also specifies that data may be available from the corresponding author upon reasonable request and after signing a data confidentiality agreement. This statement is now placed at the end of the manuscript, before the references.

Question 8: You reject the 5-class model partly because the smallest class is 4.5% (<5%). That’s a defensible heuristic, but I’d recommend phrasing it less as a hard threshold and more as: “small class size raised concerns about stability/interpretability.” Then note whether the 5-class solution was substantively meaningful or just a split of an existing class.

Response: Thank you very much for your thorough and detailed review of this manuscript. We have adopted your suggestion and revised the relevant wording in the “Latent Profile Analysis” subsection of the Results section. The revision now emphasizes that the smallest class size (4.5%) in the five-profile model raised concerns regarding the stability of parameter estimates and class interpretability. We also added a note clarifying that the five-profile solution essentially split one class from the four-profile solution into two smaller subgroups lacking clear differentiation. This revision reflects a comprehensive consideration of both statistical criteria and substantive judgment.

Question 9 : For predictors, you mention R3STEP and multinomial logistic regression. Please ensure the manuscript clearly states: which profile is the reference class, coding of predictors (some are dichotomized; grade is collapsed to Grade 3 vs Grades 4–6 in one spot—why not model grade as categorical?), whether you adjusted for clustering.

Response: We have followed your suggestion by adding clarifying information at the beginning of the “Demographic Characteristics of Latent Profiles of SEC in Elementary Students” subsection in the Results section. Specifically, we now specify the following: (1) the low level group was used as the reference class for the logistic regression; (2) the coding of all predictors (gender, only-child status, school location, and left-behind status were treated as dichotomous variables coded as 0 or 1; grade was treated as a categorical variable with Grade 3 as the reference category and dummy variables were created for Grades 4, 5, and 6); and (3) we reaffirm that robust standard errors were used to adjust for clustering at the school level in the analysis. We would like to express our sincere gratitude once again for your valuable feedback and efforts. We respectfully request your re-evaluation. If any issues remain, we greatly appreciate your further guidance.

Question 10: Because the profiles are essentially “severity levels” across all five domains, readers may wonder: did you test for more qualitatively distinct patterns (e.g., “socially strong but self-management weak”)? If your indicators are highly correlated, LPA often yields “level” classes. That’s still publishable, but you should acknowledge this and explain the practical value (screening / tiered interventions).

Response: Thank you very much for your thorough and detailed review of this manuscript. We have adopted your suggestion and added a paragraph at the end of the corresponding subsection in the Discussion section. This addition explicitly acknowledges that the profiles identified in this study primarily reflect overall level differences and elaborates on the practical significance of such “level” profiles for screening and tiered interventions. At the same time, we also note that future research could explore the possibility of “shape” profiles in more heterogeneous samples.

Question 11: Table 3 appears to have at least one formatting/labeling issue (e.g., one row seems truncated around relationship skills). Please check all tables for copyediting artifacts from the submission system.

Response: We have carefully reviewed all tables in the manuscript and corrected any potential formatting issues to ensure that the content is complete, the labels are clear, and the presentation meets journal requirements. In addition, when submitting the revised manuscript, we will upload a PDF version of the manuscript to the system for your review.

Question 12: Please tighten causal language: with cross-sectional data, statements like “SEL programs lead to…” should be framed as “may contribute” unless you directly measured program exposure or used causal designs.

Response: We have adopted your suggestion and conducted a comprehensive review and revision of the wording in the Discussion section. All expressions that could potentially be misinterpreted as implying causality have been adjusted to more cautious phrasing to align with the nature of the cross-sectional study design.

Question 13: I’d strongly encourage one short “Implications” subsection translating profiles into actions: what schools should do differently for low vs low-to-medium vs medium-to-high, whether cutoffs can be used for screening, which domains are most actionable.

Response: Thank you very much for your thorough and detailed review of this manuscript. In response to your comments, we have carefully considered and revised the manuscript accordingly. We have reorganized the “Implications for Practice” section. Based on the characteristics of the four profiles, this subsection now proposes tiered intervention strategies, discusses the possibility of setting screening thresholds based on profile proportions, and identifies the most actionable core dimensions. This revision translates the research findings into practical guidance. We would like to express our sincere gratitude once again for your valuable feedback and efforts.
