# Supplementary material for: Profiles of social-emotional competence in elementary students: A latent profile analysis
Source: PLoS One. 2026 Jun 17;21(6):e0350092. doi: 10.1371/journal.pone.0350092 (PMC13274852; doi:10.1371/journal.pone.0350092)
Supplement: Attachment — Submitted filename: Response to Reviewer.docx [file pone.0350092.s004.docx]

**Replies to the Reviewer 1**

Thank you very much for your comments on this paper. Your comments have important guidance for the improvement of this paper and our scientific research work. These pertinent comments are a great encouragement and support to us. After careful consideration and discussion, we make the following answers according to the review comments. We hope our replies and revision to the manuscript would satisfy the requirements from you. Please feel free to let me know if you have further questions on our revised manuscript. Thank you very much for your consideration and best wishes to you.

We look forward to hearing from you at your earliest convenience.

Question 1 : In the Abstract, it will be more appreciated if specific findings be introduced already (such as saying what the direction of the influences are, rather than just having a generic statement on that significant influences were observed).

Response: We are grateful to the reviewers for their insightful comments. In response to these suggestions, the abstract has been revised to include the specific directions and effect sizes (odds ratios and associated significance levels) for all demographic variables, thereby enhancing the clarity, completeness, and transparency of the reported findings.

Question 2 : Social-emotional competence (SEC) must be operationally defined first.

Response: We sincerely appreciate the reviewers for highlighting this critical issue. Consequently, the definition of SEC was inserted at its first mention, thereby satisfying the recommended requirement for operational clarity while preserving the original argumentative flow. Should further refinement of the definitional framework or additional psychometric evidence be required, the authors remain fully prepared to incorporate any supplementary revisions.

Question 3 : In the Introduction part, it has been mentioned that "few studies have examined elementary students' SEC profiles and their heterogeneous causes". It is suggested that this be expounded to include high-level summary of what the cited studies' findings are which in turn can inform the hypotheses being explored in this study.

Response: Sincerely thank you for your valuable suggestions on research innovation! In response, the extant literature has been systematically synthesized and theoretical linkages have been substantially reinforced. Specifically, (1) recent latent profile analyses have been incorporated to provide cross-grade evidence for the present hypotheses; (2) empirical indications of compensatory or imbalanced patterns among elementary school students have been elaborated; and (3) the decisive role of the synergistic development of self-regulation and social skills in the classification of SEC has been underscored. Consequently, the revised section now explicitly delineates the progressive relationship between the identified literature gap and the proposed theoretical assumptions.

Question 4 : In the sample selection, please provide more details on determining the final number of provinces/cities/schools. Are these proportional based on catchment area or population of students in official enrollment? Also, was there random sampling performed? If none, what would have been the basis of the selection of study sites? Otherwise, if there was randomization, were the responses adjusted/weighted according to the complex (multi-stage) design?

Response: Sincerely thank you for your detailed feedback! Comprehensive details of the sampling design have been added. Specifically, a probability-proportional-to-size (PPS) random-sampling method was employed to ensure representativeness and to enhance the generalizability of the findings.

Question 5 : The study reported on good reliability and structural validity of the data collection tool (questionnaire), but I wish to confirm if these reliability and validity measures were from in-sample data already, or some other study which used the same tool, or a separate pilot phase during the development part?

Response: We fully understand your rigorous requirements for data reliability. Although the Social–emotional Competencies Items represent an established instrument, all reliability indices and structural validity estimates reported herein were computed exclusively from the present sample of 11,323 valid responses. Consequently, every reliability and validity statistic reflects the current data set rather than external literature or prior samples.

Question 6 : I am curious to know how long the questionnaire is, how long it would have normally taken a respondent to response to, and would that time taken to response (say, longer than expected) could have led to "response-tiredness" which may affect data quality and "truthfulness" of the response. Was this a risk during the actual data collection? Were mitigation measures in place to address this?

Response: Thank you sincerely for your attention to the issue of questionnaire length and response fatigue. The formal questionnaire comprised 47 items, of which 40 assessed SEC on a five-point Likert scale and seven captured demographic information. The readability level was calibrated to the third-grade elementary school curriculum. During the pilot phase (n = 48), students in grades three to six completed the questionnaire in a mean time of 7.4 min, and none withdrew prematurely. In the main survey, trained administrators monitored each session; if a student exhibited hesitation or restlessness, a brief break was immediately provided. Consequently, no instance of exceeding the allotted time or abandoning the questionnaire was recorded. These observations indicate that both the questionnaire length and the required completion time were within the tolerable range for elementary school students, thereby precluding response fatigue and safeguarding the authenticity of the obtained data.

Question 7 : In Research Procedures, what is "administered in a group setting"? Kindly explain.

Response: Thank you very much for the valuable questions raised by the reviewer! The questionnaires were administered in intact classrooms during regular school hours. Thirty to forty elementary school students per class simultaneously completed the paper-and-pencil instruments under the supervision of a single examiner who had received standardized training. The examiner read the instructions aloud, demonstrated sample items, and circulated continuously to address questions, thereby ensuring procedural consistency and preventing inter-student interference.

Question 8 : On the test administrators, were they assessed post-training in terms of competence in administering the questionnaires? Were they also checked collectively if test administration was standardized? A possible concern here is that if test administration is not standard across different administrators, they may have affected the response quality as well across the different administrators.

Response: Owing to the authentic classroom setting of the present study, scheduling constraints and institutional safety regulations precluded the administration of a standardized written pre-test to examiners. To mitigate this limitation, the research team conducted two full-scale simulation sessions immediately prior to formal data collection. During these sessions, a senior researcher demonstrated the complete protocol and provided real-time corrective feedback until each examiner executed the instructions and procedures fluently and uniformly. Furthermore, every examiner received a single-page “Testing Essentials Card” that listed key instructions, standardized prompts for time management, and responses to frequently asked questions, thereby ensuring consistent verbal delivery across sessions. Although these measures cannot entirely eliminate potential variability, they represent the maximum feasible standardization within authentic instructional environments.

Question 9 : The model fitting methodology is comprehensive. May I suggest a summary diagram to make it easier to comprehend and visual?

Response: Sincerely thank you for your affirmation and valuable suggestions on our model fitting work! As shown in Table 2, the study has comprehensively reported the fit indices (AIC, BIC, aBIC, Entropy, LMR, and BLRT) that are routinely adopted and widely recognized in the international literature; these indices provide sufficient support for model selection. Upon careful consideration, the inclusion of an additional summary figure was deemed unnecessary. The rationale for this decision rests on the principles of conciseness and manuscript length: the present tabular presentation already offers a clear, numerical depiction of between-class differences, whereas an extra figure would consume further space without yielding substantive benefits.

Question 10 : In the early Results section, I may have missed it but it was not clear how the scores on the four dimensions were computed. Are these already based on the CFA, or just summed raw item scores for each dimension? I think the CFA model must have been a better option here, but just summed raw item scores for each dimension can still be valid considering the high internal consistency of the items - it is just that the underlying latent continuity of the ordinal scale per item cannot be fully established to allow the interpretability of the addition of item scores.

Response: Sincerely thank you for carefully reviewing and providing valuable suggestions! The present study derived dimensional scores through the summation of raw item responses within each dimension. This approach was adopted for three principal reasons. First, direct summation aligns with prevailing practice; extant literature consistently employs this method for analogous scales, as the reviewer correctly noted. The high homogeneity among items within each dimension ensures that summed scores remain empirically comparable across studies. Second, the exceptional internal consistency of the five dimensions (Cronbach’s α > 0.75) substantiates the validity of additive aggregation. Although confirmatory factor analysis (CFA) more precisely captures latent structures, the observed minimal inter-item variability indicates that simple summation robustly represents dimensional levels. Third, the parsimony of the additive method enhances interpretability and practical applicability. By circumventing potential model-misfit issues inherent in CFA, summed scores are readily comprehensible to readers and can be directly employed for evidence-based decisions.

Question 11 : I suggest formally defining or providing an operational definition of "left-behind".

Response: We are sincerely grateful for your recommendation to provide an explicit definition of “left-behind children.” Accordingly, a clear and quantifiable definition has been added to ensure conceptual clarity and to facilitate subsequent empirical comparisons and policy analyses.

Question 12 : I note the ultimate choice of considering only 4 latent profiles due to class size issues, but I wonder if the differences in the suggested profile were also checked in this 5-class model? It appears reducing the number of classes does not simply result to redistributing the "small" class to another class dominantly, so it may either mean that the class/profile structure may be very different between the 4- and 5-class models, or that the redistributed class is heterogenous with respect to the classes in the 4-class model? In general, I would have been interested into deep diving the characteristics of the resulting classes/profiles, beyond just high - to - low SEC scores.

Response: We are sincerely grateful for your meticulous examination of the latent class analysis results and for their highly constructive insights. The concerns regarding the potential structural differences between the four and five class solutions, as well as the need for a deeper exploration of class characteristics, have been recognized as pivotal to the justification of model selection.

First, regarding the between-class differences within the five class model, the authors conducted a thorough inspection during model fitting. Nevertheless, the smallest class in this solution comprised only 4.5% of the sample, falling below the established 5% threshold. This limitation significantly increases parameter-estimation uncertainty and overall model instability. Moreover, the between-class differences in the five-class model did not reveal any qualitatively distinct dimensions beyond the continuous low-to-high continuum. Specifically, across the five core dimensions—self-awareness, self-management, social awareness, relationship skills, and responsible decision-making—the additional class merely represented a minor subdivision within either the high or low scoring segment, rather than introducing a substantively new profile characterized by atypical performance or a unique deficit pattern.

Consequently, the four-class model was retained for the following reasons. (1) The minimum class size criterion was strictly applied to ensure robust parameter estimates and model stability. (2) The four-class solution achieved an optimal balance between information-criterion evidence (a plateau in AIC, BIC, and aBIC reductions) and substantive interpretability. (3) Most critically, the four-class model captures the principal variation in SEC along a coherent low-to-high continuum across all five dimensions, thereby exhibiting both statistical significance and clear psychological and educational relevance.

The authors fully concur that an in-depth characterization of each profile is essential for elucidating population heterogeneity. Accordingly, the Discussion section has been expanded to provide a more nuanced description and interpretation of the specific score patterns exhibited by each latent class across the five dimensions.

Collectively, the empirical evidence and the study objectives indicate that the four-class model, labeled in terms of the low, low to medium, medium to high, and high level groups, represents the most appropriate, robust, and interpretable solution. Therefore, the authors respectfully propose that the current analytical framework and associated descriptions remain unchanged, as they adequately and accurately reflect the core findings.

Question 13 : On section 3.4, these findings are expected anyway because of the exploratory analyses conducted simply comparing SEC scores by sociodemographic profile. I would have been more interested to see if there were important "deviations" in expected classifications into profiles. For example, who are those males which have been classified in the High SEC profile? What is with them that raised their SEC, etc. This actually relates to one of your limitation (#2) on looking at the interactions between variables affecting SEC. While I understand this is not a formal consideration now, perhaps you may want to make a crude analysis on the associations between (using pairwise Chi-Sqaure Test of Associations) your variables, to see for example if there were more Females in the Urban region that could have resulted to for both categories, SEC was raised (so there is confounding or mediation).

We are sincerely appreciate the reviewers’ meticulous review and invaluable feedback. The suggestion to explore “deviant” cases within the latent profiles of SEC and to investigate potential interactive effects between variables is highly instructive and perceptively addresses the limitations noted in our manuscript. We fully recognize the reviewers’ interest in these underlying mechanisms, which represent a valuable avenue for future inquiry. Here, we respectfully provide clarifications regarding the specific comments on Section 3.4.

First, the primary objective of the present study was to systematically delineate the overall developmental patterns of SEC among elementary school students in China and to establish their preliminary associations with key demographic characteristics. Latent profile analysis was employed to provide a data-driven, objective method for identifying heterogeneous subgroups, thereby avoiding the subjectivity inherent in a priori classifications. Subsequently, multinomial logistic regression served as a direct and widely validated tool for quantitatively assessing the influence of demographic variables on the probability of individual membership in each SEC category. This analytical framework has been extensively adopted in comparable studies and has proven effective in addressing the central research question: “Which background characteristics increase the likelihood of students belonging to a specific developmental pattern?” Although several associations were anticipated, the descriptive results presented constitute foundational evidence that clarifies the current status of SEC development among Chinese elementary school students. These findings enable the identification of potential risk or protective groups and carry important policy implications, thereby establishing a basis for more refined mechanistic investigations.

Second, we concur that an examination of “anomalous” cases and potential confounding or interactive effects would substantially enrich the depth of the analysis. Indeed, preliminary analyses were conducted to address this consideration. Individual-level data within the high level group were carefully inspected, with particular attention to male students, in an attempt to identify distinctive characteristics or background factors that might systematically differentiate these individuals from their counterparts in either the high level group or the low level group. Regrettably, the variables available in the current dataset did not reveal any consistently influential “key factors” or unique “profiles.” These students exhibited considerable heterogeneity across all measured indicators and background variables, precluding the identification of a coherent and distinctive pattern.

With regard to possible interactions or confounding among variables, exploratory analyses were also undertaken. Chi-square tests were employed to examine pairwise associations among key demographic variables. Preliminary results indicated modest associations—for example, urban schools displayed a slightly higher proportion of only children; however, most associations were weak. More importantly, when two-way interaction terms were added to the multinomial logistic model or when stratified analyses were conducted (e.g., examining gender effects separately within urban and rural subsamples), no significant or consistent interactions emerged, and the patterns observed were aligned with those in the overall sample. Owing to the limited sample size in certain subgroups and the absence of a priori power calculations for interaction effects, these supplementary analyses are subject to multiple-comparison concerns and should be interpreted cautiously. Consequently, they did not yield sufficiently robust or novel insights.

In summary, the results and analyses presented in Section 3.4 have achieved their intended purpose: to provide a clear exposition of the associations between key demographic backgrounds and distinct SEC developmental patterns. We remain fully cognizant of the reviewers’ desire for more nuanced analyses, and we will explicitly emphasize in the limitations section that the exploration of individual trait heterogeneity and interactive effects constitutes an important yet unresolved issue. Such investigations will require future studies adopting more sophisticated longitudinal designs or experimental interventions.

Question 14 : Was ordinal regression considered, noting the natural ordinality with respect to SEC scores of the resultant profiles in assessing the associations with demographic characteristics (section 3.4)? I think even a proportional odds model assumption would suffice considering the exploratory nature of the analysis. It is because I think that some (statistical) information may be lost by not considering the ordinality of the SEC scores which define the profiles?

Response: Thank you sincerely for your valuable suggestion on whether to adopt ordered regression. Multinomial logistic regression was selected on the basis of the following considerations. First, the primary objective of the present study was to identify and characterize latent classes of social emotional competence (SEC) among elementary school students rather than to impose an a priori ordering on the levels of competence. Multinomial logistic regression simultaneously estimates the independent effects of each class relative to a reference category, a procedure that aligns with established practices in exploratory categorical analyses and has been widely adopted in comparable SEC investigations.

Second, the proportional odds assumption was formally evaluated following the reviewers’ guidance. Brant’s test yielded p < 0.05, thereby rejecting the parallel-lines assumption. Subsequent application of a partial proportional odds model revealed that only two covariates satisfied the assumption, whereas the remainder did not. Moreover, the model-fit indices (AIC and BIC) did not indicate a significant improvement over the multinomial logistic model. Consequently, ordinal regression was deemed inappropriate for the present sample; its adoption would likely introduce estimation bias. The current analytical approach therefore more effectively serves the objective of elucidating heterogeneous influences.

Question 15 : Please correct me if I am wrong, but I may have been confused with the "only child" findings. The descriptive statistics (Table 1) indicate that only children scored significantly higher on all five SEC dimensions compared to non-only children. However, the multinomial logistic regression (Table 5) and the subsequent discussion report that non-only children were more likely to be in higher SEC groups?

Response: First of all, I sincerely thank you for your rigorous review of the details of the paper. Regarding the ostensible contradiction between family structure and social emotional competence (SEC), further verification by the research team has confirmed statistical coherence. Independent-samples t tests indicated that only children obtained higher raw SEC scores on all five dimensions, thereby reflecting absolute between-group differences in ability. Conversely, the multinomial logistic regression classified participants into the high level group on the basis of predicted probabilities of group membership; its focus was on the likelihood of an individual being assigned to the high ability stratum. Owing to the substantially larger base rate of non-only children (80.45 % of the sample), extreme value distributions within relative rankings were more probable. Should additional clarification be required.

**Replies to the Reviewer 2**

Thank you very much for your comments on this paper. Your comments have important guidance for the improvement of this paper and our scientific research work. These pertinent comments are a great encouragement and support to us. After careful consideration and discussion, we make the following answers according to the review comments. We hope our replies and revision to the manuscript would satisfy the requirements from you. Please feel free to let me know if you have further questions on our revised manuscript. Thank you very much for your consideration and best wishes to you.

We look forward to hearing from you at your earliest convenience.

Question 1: Strengthen the Literature Review and Research Gap

The introduction cites relevant frameworks (CASEL, OECD, and Chinese SEC model) but lacks a synthesized literature review on:

a) Prior LPA or PCA studies in SEC or related constructs among children, and

b) Why the current sample, variables, and context make this study unique.

Explicitly state the research gap and contribution to the literature.

Response: We sincerely thank you for the valuable suggestion to strengthen the literature review and to clarify the research gap. In response, two principal revisions have been implemented. First, additional literature concerning the latent profiles of SEC has been incorporated to establish a robust theoretical foundation. Second, the unique contribution of the present study has been articulated through the following three points: (1) a paucity of research examining the developmental characteristics of SEC among Chinese elementary school students; (2) the predominant focus of existing latent profile analysis (LPA) on adolescent populations, with a notable absence of investigations at the elementary level; and (3) the insufficient elucidation of the mechanisms through which demographic variables—specifically gender and grade—shape the distribution of SEC subtypes.

Question 2: Clarify Theoretical Underpinning

While multiple models of SEC are referenced, the manuscript does not clearly articulate a guiding theoretical framework.

Define how theory informs:

a) The selection of SEC dimensions,

b) Expectations for latent profiles, and

c) Hypotheses regarding demographic predictors.

Response: Thank you very much for your valuable feedback. We have carefully revised the manuscript based on your suggestions. First, the theoretical foundation of the Collaborative for Academic, Social, and Emotional Learning (CASEL) framework has been explicitly delineated, and the principal reasons for adopting this framework have been clarified. Second, clarification is provided regarding the absence of specific a priori expectations for the latent profiles. Latent profile analysis (LPA) is inherently an exploratory data-analytic technique whose strength lies in allowing the class structure to emerge from the data rather than in verifying predefined theoretical taxonomies. Consequently, in accordance with established methodological conventions, no prior hypotheses were formulated concerning the number or characteristics of the latent profiles, thereby ensuring the objectivity of the analytical outcomes. Furthermore, demographic variables were conceptualized as covariates rather than core predictors. Subsequent to the completion of the LPA, the BCH procedure was employed to examine distributional differences of these covariates across the identified profiles. This “classification-then-comparison” sequence represents the standard analytical workflow for incorporating covariates within latent class models, and its purpose is to provide descriptive insights into between-group differences rather than to test causal hypotheses. It is anticipated that this exploratory approach will generate valuable findings for future investigations while avoiding unwarranted theoretical speculation.

Question 3: Justify the Person-Centered Approach (LPA)

Currently, the rationale for LPA is generic and could apply to any study of heterogeneity.

Please provide:

a) A stronger argument for why LPA is superior to traditional variable-centered approaches in this context,

b) Citations to related LPA studies in SEC, social skills, or educational psychology, and

c) Discussion of how your profiles are conceptually meaningful beyond descriptive clustering.

Response: Thank you for accurately pointing out the issue In response, three clarifications have been introduced. First, recent theoretical and empirical literature has been incorporated to explicate the operating principles and objectives of LPA, thereby demonstrating its appropriateness for research on SEC. Second, the multidimensional and interactive nature of SEC has been accentuated; this non-linear configuration aligns precisely with the patterns that LPA is designed to capture. Third, the identification of SEC subgroups has been elaborated as pivotal evidence for understanding the complexity and unequal development trajectories of SEC, thereby providing a theoretical foundation for differentiated interventions. Consequently, the revised section now unequivocally establishes the irreplaceable role of LPA in SEC research.

Question 4: Align Results with the Person-Centered Approach

The manuscript devotes substantial space to t-tests and ANOVA on SEC dimensions across demographics (a variable-centered approach).

These results partially defeat the purpose of adopting LPA. I recommend:

a) Presenting such analyses as brief descriptive context or in supplementary material, and

b) Focusing the main results and discussion on LPA-derived profiles and their demographic predictors.

Response: Thank you sincerely for your important suggestions on the methodology of the paper. In full agreement with the necessity of foregrounding a person-centered analytic framework, the descriptive statistics section and its associated tables have been removed. Consequently, the Results section now contains only the latent profile analysis and the subsequent multinomial logistic regression, thereby ensuring that the manuscript remains consistently aligned with the person-centered perspective. This streamlining renders the manuscript more concise without compromising data transparency; the complete descriptive statistics remain available upon reasonable request.

Question 5: Elaborate on Practical and Policy Implications

The “Practical Implications” section should clearly explain:

a) Why LPA-based profiles offer actionable insights for tailored interventions,

b) How schools, parents, or policymakers can use profile information differently than mean-based approaches, and

c) Examples of how educational or developmental interventions could be profile-specific.

Response: We express their sincere gratitude for the insightful recommendations regarding the “Practical Implications” section. In response, the manuscript has been revised to clarify the intervention advantages derived from latent profile analysis (LPA) through three refinements. First, the distinctive value of LPA classification over conventional mean-based analyses has been explicitly emphasized. Second, differentiated intervention examples tailored to each identified profile have been provided. Third, concrete and actionable implementation pathways have been delineated. These revisions substantially enhance the practical guidance of the study.

Question 6: Ethics and Child Assent

While parental consent is mentioned, the manuscript does not specify:

a) Whether child assent was obtained,

b) How psychological risks (e.g., stress or discomfort from questions) were mitigated.

PLOS ONE requires explicit reporting for research with minors.

Response: We gratefully acknowledge your meticulous attention to the ethical details of this study. In accordance with the actual research procedures and the reviewers’ recommendations, the following clarifications have been incorporated into the “Research Procedure” subsection to enhance transparency and to safeguard participant rights.

First, a standardized informed consent form was developed before questionnaire construction. Given the limited reading proficiency and time constraints of elementary school students, the lead researcher orally presented the contents of the form to the entire class. After the explanation, each student independently marked either “I consent to participate” or “I do not consent.”

Second, potential psychological burden was minimized through preventive measures. A pilot test involving 48 elementary school students indicated that the questionnaire content and administration duration were well tolerated; no participant withdrew, and no signs of psychological fatigue were observed. Moreover, prior to data collection, the lead researcher explicitly described the voluntary withdrawal procedure, and students were informed that they could discontinue their participation at any time without penalty.

These procedural details have now been fully documented in the revised manuscript.

Question 7: Temper Developmental Claims

As the design is cross-sectional, any claims of “development” with grade level should be reframed as grade differences rather than longitudinal growth.

Response: We are deeply grateful for the reviewers’ meticulous comments on methodological rigor. In full agreement with the observation that cross-sectional data cannot support longitudinal developmental inferences, the manuscript has been revised so that all references to “development” have been replaced with “grade-level differences.” A comprehensive review of the entire text was conducted to eliminate any wording implying longitudinal change. Moreover, the Discussion section now explicitly acknowledges the inherent limitations of the cross-sectional design. These revisions substantially enhance the scientific integrity of the work.
