# Supplementary material for: Profiles of social-emotional competence in elementary students: A latent profile analysis
Source: PLoS One. 2026 Jun 17;21(6):e0350092. doi: 10.1371/journal.pone.0350092 (PMC13274852; doi:10.1371/journal.pone.0350092)
Supplement: Attachment — Submitted filename: Response_to_Reviewers_auresp_5.docx [file pone.0350092.s006.docx]

**Replies to the Reviewer**

Thank you very much for your comments on this paper. Your comments have important guidance for the improvement of this paper and our scientific research work. These pertinent comments are a great encouragement and support to us. After careful consideration and discussion, we make the following answers according to the review comments. We hope our replies and revision to the manuscript would satisfy the requirements from you. Please feel free to let me know if you have further questions on our revised manuscript. Thank you very much for your consideration and best wishes to you.

We look forward to hearing from you at your earliest convenience.

***Question 1:*** Please carefully recheck the reported odds ratios in the Abstract and Table 4. Some values reported in the Abstract as odds ratios appear to correspond to the regression coefficients in Table 4 rather than the OR values. Please verify these against the original Mplus output and ensure that the Abstract, Table 4, and response to reviewers are fully consistent.

***Response:*** Thank you very much for your thorough review of our manuscript and for your keen observation that some odds ratios (ORs) in the abstract might have been confused with regression coefficients. In response to your comment, we rechecked the original Mplus output and corrected each OR value in the abstract and Table 4, ensuring that all reported values are correct ORs rather than regression coefficients. Moreover, we have standardized the relevant data across the abstract, the table, and the response letter to the reviewer, guaranteeing full consistency throughout the manuscript.

***Question 2:*** Please further soften the remaining causal language in the manuscript, given the cross-sectional design. Phrases suggesting influence, effects, or mechanisms should be revised to associative wording, such as “were linked to profile membership,” “were more likely to belong to specific profiles,” or “were associated with SEC profiles.”

***Response:*** Your rigorous attitude has been highly instructive for improving the academic rigor of our manuscript. We have revised all expressions that could imply causal inference (e.g., “influence,” “effects,” “mechanisms,” “enhance,” “exacerbate”) to associative wording such as “were associated with,” “may be related to,” or “were linked to profile membership,” ensuring that the language aligns with the cross-sectional study design. It should be noted that some causal phrasing appears in the cited previous studies (e.g., “Studies show that … lead to …”). Out of respect for the findings of the original literature and in compliance with academic norms, we did not alter the original wording within those citations. However, we have clearly indicated the sources of these citations in the text, and all conclusion statements from our own study have been expressed using associative language, so no causal misinterpretation should arise from the overall context.

We believe the above revisions have addressed your concerns. If any further adjustments are needed, we respectfully request that you specify the particular passage or sentence (e.g., by page number, paragraph, and line number) to enable us to make precise revisions. Vague general comments are not conducive to our effective response to your concerns. Thank you again for your understanding and support.

***Question 3:*** please ensure that all tracked changes are accepted in the clean version of the manuscript, unless a tracked version is specifically required. This will help avoid the appearance that previously revised terms, such as “subtype,” remain in the manuscript.

***Response:*** Your suggestion has been very important for ensuring the clarity and standardization of our manuscript. In the final clean version of the manuscript, we have accepted all previous revisions (including terminology unification, wording modifications, and table corrections), ensuring that no track changes remain visible. This avoids potential misunderstandings that could arise from residual revision marks (e.g., strikethrough or markup of previously revised terms such as “subtype”). After accepting all revisions, we rechecked the entire manuscript and confirmed that all instances of “subtype” or “subtypes” have been replaced with “profile” or “profile membership,” and that the wording throughout the manuscript is fully consistent with the final revised version.

***Question 4:*** Please confirm that the Data Availability Statement is identical across the manuscript, cover letter, and submission form, including the ethics committee name, contact email, access criteria, and the requirement for a data confidentiality agreement.

***Response:*** Thank you very much for reviewing our manuscript again. In response to your comment, we have standardized the Data Availability Statement across the manuscript, the cover letter, and the submission form, ensuring that the content is identical in all three places. All versions explicitly include the full name of the ethics committee, a valid contact email address, the specific criteria for data access, and the requirement to sign a data confidentiality agreement.
