# Supplementary material for: Profiles of social-emotional competence in elementary students: A latent profile analysis
Source: PLoS One. 2026 Jun 17;21(6):e0350092. doi: 10.1371/journal.pone.0350092 (PMC13274852; doi:10.1371/journal.pone.0350092)
Supplement: Peer review history

# Peer review: Decision Letter 0

**DOI:** 10.1371/journal.pone.0350092.r001

**Authors:** Zypher Jude Regencia

4 Aug 2025

PONE-D-25-19637

Subtypes of Social-Emotional Competence in Elementary Students: A Latent Profile Analysis

PLOS ONE

Dear Dr. Wang,

Thank you for submitting your manuscript to PLOS ONE. After careful consideration, we feel that it has merit but does not fully meet PLOS ONE’s publication criteria as it currently stands. Therefore, we invite you to submit a revised version of the manuscript that addresses the points raised during the review process.

Please submit your revised manuscript by Sep 18 2025 11:59PM. If you will need more time than this to complete your revisions, please reply to this message or contact the journal office at plosone@plos.org. When you're ready to submit your revision, log on to https://www.editorialmanager.com/pone/ and select the 'Submissions Needing Revision' folder to locate your manuscript file.

Please include the following items when submitting your revised manuscript:

- A rebuttal letter that responds to each point raised by the academic editor and reviewer(s). You should upload this letter as a separate file labeled 'Response to Reviewers'.
- A marked-up copy of your manuscript that highlights changes made to the original version. You should upload this as a separate file labeled 'Revised Manuscript with Track Changes'.
- An unmarked version of your revised paper without tracked changes. You should upload this as a separate file labeled 'Manuscript'.

If you would like to make changes to your financial disclosure, please include your updated statement in your cover letter. Guidelines for resubmitting your figure files are available below the reviewer comments at the end of this letter.

If applicable, we recommend that you deposit your laboratory protocols in protocols.io to enhance the reproducibility of your results. Protocols.io assigns your protocol its own identifier (DOI) so that it can be cited independently in the future. For instructions see: https://journals.plos.org/plosone/s/submission-guidelines#loc-laboratory-protocols. Additionally, PLOS ONE offers an option for publishing peer-reviewed Lab Protocol articles, which describe protocols hosted on protocols.io. Read more information on sharing protocols at https://plos.org/protocols?utm_medium=editorial-email&utm_source=authorletters&utm_campaign=protocols.

We look forward to receiving your revised manuscript.

Kind regards,

Zypher Jude G. Regencia, Ph.D.

Academic Editor

PLOS ONE

Journal requirements:

When submitting your revision, we need you to address these additional requirements.

1. Please ensure that your manuscript meets PLOS ONE's style requirements, including those for file naming. The PLOS ONE style templates can be found at

https://journals.plos.org/plosone/s/file?id=wjVg/PLOSOne_formatting_sample_main_body.pdf and

https://journals.plos.org/plosone/s/file?id=ba62/PLOSOne_formatting_sample_title_authors_affiliations.pdf

2. Thank you for stating the following financial disclosure:

“Xiang Gao is supported by the National Office for Education Sciences Planning (CLA230300).”

Please state what role the funders took in the study.  If the funders had no role, please state: "The funders had no role in study design, data collection and analysis, decision to publish, or preparation of the manuscript."

If this statement is not correct you must amend it as needed.

Please include this amended Role of Funder statement in your cover letter; we will change the online submission form on your behalf.

3. Thank you for stating the following in the Acknowledgments Section of your manuscript:

“This research was funded by the National Office for Education Sciences Planning (CLA230300).”

We note that you have provided funding information that is currently declared in your Funding Statement. However, funding information should not appear in the Acknowledgments section or other areas of your manuscript. We will only publish funding information present in the Funding Statement section of the online submission form.

Please remove any funding-related text from the manuscript and let us know how you would like to update your Funding Statement. Currently, your Funding Statement reads as follows:

“Xiang Gao is supported by the National Office for Education Sciences Planning (CLA230300).”

Please include your amended statements within your cover letter; we will change the online submission form on your behalf.

4. We note that you have indicated that there are restrictions to data sharing for this study. PLOS only allows data to be available upon request if there are legal or ethical restrictions on sharing data publicly. For more information on unacceptable data access restrictions, please see http://journals.plos.org/plosone/s/data-availability#loc-unacceptable-data-access-restrictions.

Before we proceed with your manuscript, please address the following prompts:

a) If there are ethical or legal restrictions on sharing a de-identified data set, please explain them in detail (e.g., data contain potentially identifying or sensitive patient information, data are owned by a third-party organization, etc.) and who has imposed them (e.g., a Research Ethics Committee or Institutional Review Board, etc.). Please also provide contact information for a data access committee, ethics committee, or other institutional body to which data requests may be sent.

b) If there are no restrictions, please upload the minimal anonymized data set necessary to replicate your study findings to a stable, public repository and provide us with the relevant URLs, DOIs, or accession numbers. For a list of recommended repositories, please see

https://journals.plos.org/plosone/s/recommended-repositories. You also have the option of uploading the data as Supporting Information files, but we would recommend depositing data directly to a data repository if possible.

We will update your Data Availability statement on your behalf to reflect the information you provide.

If the reviewer comments include a recommendation to cite specific previously published works, please review and evaluate these publications to determine whether they are relevant and should be cited. There is no requirement to cite these works unless the editor has indicated otherwise.

Reviewers' comments:

Reviewer's Responses to Questions

**Comments to the Author**

1. Is the manuscript technically sound, and do the data support the conclusions?

The manuscript must describe a technically sound piece of scientific research with data that supports the conclusions. Experiments must have been conducted rigorously, with appropriate controls, replication, and sample sizes. The conclusions must be drawn appropriately based on the data presented.

Reviewer #1: Yes

Reviewer #2: Partly

**********

2. Has the statistical analysis been performed appropriately and rigorously?

Reviewer #1: Yes

Reviewer #2: No

**********

3. Have the authors made all data underlying the findings in their manuscript fully available?

The PLOS Data policy requires authors to make all data underlying the findings described in their manuscript fully available without restriction, with rare exception (please refer to the Data Availability Statement in the manuscript PDF file). The data should be provided as part of the manuscript or its supporting information, or deposited to a public repository. For example, in addition to summary statistics, the data points behind means, medians and variance measures should be available. If there are restrictions on publicly sharing data—e.g. participant privacy or use of data from a third party—those must be specified.

Reviewer #1: No

Reviewer #2: Yes

**********

4. Is the manuscript presented in an intelligible fashion and written in standard English?

PLOS ONE does not copyedit accepted manuscripts, so the language in submitted articles must be clear, correct, and unambiguous. Any typographical or grammatical errors should be corrected at revision, so please note any specific errors here.

Reviewer #1: Yes

Reviewer #2: Yes

**********

5. Review Comments to the Author

Please use the space provided to explain your answers to the questions above. You may also include additional comments for the author, including concerns about dual publication, research ethics, or publication ethics. (Please upload your review as an attachment if it exceeds 20,000 characters)

Reviewer #1: Thank you for the opportunity to review the manuscript titled "Subtypes of Social-Emotional Competence in Elementary Students: A Latent Profile Analysis". The study investigated to identify distinct subgroups (latent profiles) of elementary school students in China based on their social-emotional competence (SEC) and to examine how demographic variables predict membership in these profiles. This manuscript has the potential to make a valuable contribution to the literature on SEC in elementary students. The study's strengths, particularly its large sample size and geographically diverse sample, as well as the use of a sound statistical analysis framework especially with the use of LPA, are commendable. There are a few questions that I would like to seek clarification with (which I detail below). Nevertheless, the merits outweigh my perceived limitations; therefore, this manuscript has my full recommendation for publication contingent on satisfactory revisions and responses to my specific queries, comments, and suggestions below.

Comments / Suggestions / Questions:

1.) In the Abstract, it will be more appreciated if specific findings be introduced already (such as saying what the direction of the influences are, rather than just having a generic statement on that significant influences were observed).

2.) Social-emotional competence (SEC) must be operationally defined first.

3.) In the Introduction part, it has been mentioned that "few studies have examined elementary students'

SEC profiles and their heterogeneous causes". It is suggested that this be expounded to include high-level summary of what the cited studies' findings are which in turn can inform the hypotheses being explored in this study.

4.) In the sample selection, please provide more details on determining the final number of provinces/cities/schools. Are these proportional based on catchment area or population of students in official enrollment? Also, was there random sampling performed? If none, what would have been the basis of the selection of study sites? Otherwise, if there was randomization, were the responses adjusted/weighted according to the complex (multi-stage) design?

5.) The study reported on good reliability and structural validity of the data collection tool (questionnaire), but I wish to confirm if these reliability and validity measures were from in-sample data already, or some other study which used the same tool, or a separate pilot phase during the development part?

6.) I am curious to know how long the questionnaire is, how long it would have normally taken a respondent to response to, and would that time taken to response (say, longer than expected) could have led to "response-tiredness" which may affect data quality and "truthfulness" of the response. Was this a risk during the actual data collection? Were mitigation measures in place to address this?

7.) In Research Procedures, what is "administered in a group setting"? Kindly explain.

8.) On the test administrators, were they assessed post-training in terms of competence in administering the questionnaires? Were they also checked collectively if test administration was standardized? A possible concern here is that if test administration is not standard across different administrators, they may have affected the response quality as well across the different administrators.

9.) The model fitting methodology is comprehensive. May I suggest a summary diagram to make it easier to comprehend and visual?

10.) In the early Results section, I may have missed it but it was not clear how the scores on the four dimensions were computed. Are these already based on the CFA, or just summed raw item scores for each dimension? I think the CFA model must have been a better option here, but just summed raw item scores for each dimension can still be valid considering the high internal consistency of the items - it is just that the underlying latent continuity of the ordinal scale per item cannot be fully established to allow the interpretability of the addition of item scores.

11.) I suggest formally defining or providing an operational definition of "left-behind".

12.) I note the ultimate choice of considering only 4 latent profiles due to class size issues, but I wonder if the differences in the suggested profile were also checked in this 5-class model? It appears reducing the number of classes does not simply result to redistributing the "small" class to another class dominantly, so it may either mean that the class/profile structure may be very different between the 4- and 5-class models, or that the redistributed class is heterogenous with respect to the classes in the 4-class model? In general, I would have been interested into deep diving the characteristics of the resulting classes/profiles, beyond just high - to - low SEC scores.

13.) On section 3.4, these findings are expected anyway because of the exploratory analyses conducted simply comparing SEC scores by sociodemographic profile. I would have been more interested to see if there were important "deviations" in expected classifications into profiles. For example, who are those males which have been classified in the High SEC profile? What is with them that raised their SEC, etc. This actually relates to one of your limitation (#2) on looking at the interactions between variables affecting SEC. While I understand this is not a formal consideration now, perhaps you may want to make a crude analysis on the associations between (using pairwise Chi-Sqaure Test of Associations) your variables, to see for example if there were more Females in the Urban region that could have resulted to for both categories, SEC was raised (so there is confounding or mediation).

14.) Was ordinal regression considered, noting the natural ordinality with respect to SEC scores of the resultant profiles in assessing the associations with demographic characteristics (section 3.4)? I think even a proportional odds model assumption would suffice considering the exploratory nature of the analysis. It is because I think that some (statistical) information may be lost by not considering the ordinality of the SEC scores which define the profiles?

15.) Please correct me if I am wrong, but I may have been confused with the "only child" findings. The descriptive statistics (Table 1) indicate that only children scored significantly higher on all five SEC dimensions compared to non-only children. However, the multinomial logistic regression (Table 5) and the subsequent discussion report that non-only children were more likely to be in higher SEC groups?

Reviewer #2: The manuscript addresses an important topic in social-emotional competence (SEC) among elementary students and employs a person-centered analytic approach (Latent Profile Analysis, LPA). While the study is based on a large and diverse sample, the manuscript currently requires substantial revision to improve its conceptual framing, methodological justification, and alignment between aims and analyses. Below, I outline key areas that require attention.

Revisions Required

1. Strengthen the Literature Review and Research Gap

The introduction cites relevant frameworks (CASEL, OECD, and Chinese SEC model) but lacks a synthesized literature review on:

a) Prior LPA or PCA studies in SEC or related constructs among children, and

b) Why the current sample, variables, and context make this study unique.

Explicitly state the research gap and contribution to the literature.

2. Clarify Theoretical Underpinning

While multiple models of SEC are referenced, the manuscript does not clearly articulate a guiding theoretical framework.

Define how theory informs:

a) The selection of SEC dimensions,

b) Expectations for latent profiles, and

c) Hypotheses regarding demographic predictors.

3. Justify the Person-Centered Approach (LPA)

Currently, the rationale for LPA is generic and could apply to any study of heterogeneity.

Please provide:

a) A stronger argument for why LPA is superior to traditional variable-centered approaches in this context,

b) Citations to related LPA studies in SEC, social skills, or educational psychology, and

c) Discussion of how your profiles are conceptually meaningful beyond descriptive clustering.

3. Align Results with the Person-Centered Approach

The manuscript devotes substantial space to t-tests and ANOVA on SEC dimensions across demographics (a variable-centered approach).

These results partially defeat the purpose of adopting LPA. I recommend:

a) Presenting such analyses as brief descriptive context or in supplementary material, and

b) Focusing the main results and discussion on LPA-derived profiles and their demographic predictors.

4. Elaborate on Practical and Policy Implications

The “Practical Implications” section should clearly explain:

a) Why LPA-based profiles offer actionable insights for tailored interventions,

b) How schools, parents, or policymakers can use profile information differently than mean-based approaches, and

c) Examples of how educational or developmental interventions could be profile-specific.

5. Ethics and Child Assent

While parental consent is mentioned, the manuscript does not specify:

a) Whether child assent was obtained,

b) How psychological risks (e.g., stress or discomfort from questions) were mitigated.

PLOS ONE requires explicit reporting for research with minors.

6. Temper Developmental Claims

As the design is cross-sectional, any claims of “development” with grade level should be reframed as grade differences rather than longitudinal growth.

This is a potentially valuable contribution on elementary students’ SEC subtypes in China. To reach publishable quality, the manuscript needs clearer theoretical grounding, stronger justification for LPA, alignment between aims and methods, and explicit reporting of ethics and data transparency. I recommend major revisions before the manuscript can be considered for publication.

**********

6. PLOS authors have the option to publish the peer review history of their article (what does this mean?). If published, this will include your full peer review and any attached files.

If you choose “no”, your identity will remain anonymous but your review may still be made public.

**Do you want your identity to be public for this peer review?** For information about this choice, including consent withdrawal, please see our Privacy Policy.

Reviewer #1: No

Reviewer #2: No

While revising your submission, please upload your figure files to the Preflight Analysis and Conversion Engine (PACE) digital diagnostic tool, https://pacev2.apexcovantage.com/. PACE helps ensure that figures meet PLOS requirements. To use PACE, you must first register as a user. Registration is free. Then, login and navigate to the UPLOAD tab, where you will find detailed instructions on how to use the tool. If you encounter any issues or have any questions when using PACE, please email PLOS at figures@plos.org. Please note that Supporting Information files do not need this step.

# Author comment: Author response to Decision Letter 1

**DOI:** 10.1371/journal.pone.0350092.r002

30 Aug 2025

Thank you very much for your comments on this paper. Your comments have important guidance for the improvement of this paper and our scientific research work. These pertinent comments are a great encouragement and support to us. After careful consideration and discussion, we make the following answers according to the review comments. We hope our replies and revision to the manuscript would satisfy the requirements from you. Please feel free to let me know if you have further questions on our revised manuscript. Thank you very much for your consideration and best wishes to you.

We look forward to hearing from you at your earliest convenience.

Attachment Submitted filename: Response to Reviewers.docx

# Peer review: Decision Letter 1

**DOI:** 10.1371/journal.pone.0350092.r003

**Authors:** Zypher Jude Regencia

6 Feb 2026

PONE-D-25-19637R1

Subtypes of Social-Emotional Competence in Elementary Students: A Latent Profile Analysis

PLOS One

Dear Dr. Wang,

Thank you for submitting your manuscript to PLOS ONE. After careful consideration, we feel that it has merit but does not fully meet PLOS ONE’s publication criteria as it currently stands. Therefore, we invite you to submit a revised version of the manuscript that addresses the points raised during the review process. Since there are no available reviewers, I have made a decision to review the manuscript.

Please submit your revised manuscript by Mar 22 2026 11:59PM. If you will need more time than this to complete your revisions, please reply to this message or contact the journal office at plosone@plos.org. When you're ready to submit your revision, log on to https://www.editorialmanager.com/pone/ and select the 'Submissions Needing Revision' folder to locate your manuscript file.

Please include the following items when submitting your revised manuscript:

- A letter that responds to each point raised by the academic editor and reviewer(s). You should upload this letter as a separate file labeled 'Response to Reviewers'.
- A marked-up copy of your manuscript that highlights changes made to the original version. You should upload this as a separate file labeled 'Revised Manuscript with Track Changes'.
- An unmarked version of your revised paper without tracked changes. You should upload this as a separate file labeled 'Manuscript'.

If you would like to make changes to your financial disclosure, please include your updated statement in your cover letter. Guidelines for resubmitting your figure files are available below the reviewer comments at the end of this letter.

If applicable, we recommend that you deposit your laboratory protocols in protocols.io to enhance the reproducibility of your results. Protocols.io assigns your protocol its own identifier (DOI) so that it can be cited independently in the future. For instructions see: https://journals.plos.org/plosone/s/submission-guidelines#loc-laboratory-protocols. Additionally, PLOS ONE offers an option for publishing peer-reviewed Lab Protocol articles, which describe protocols hosted on protocols.io. Read more information on sharing protocols at https://plos.org/protocols?utm_medium=editorial-email&utm_source=authorletters&utm_campaign=protocols.

We look forward to receiving your revised manuscript.

Kind regards,

Zypher Jude G. Regencia, Ph.D.

Academic Editor

PLOS One

Journal Requirements:

If the reviewer comments include a recommendation to cite specific previously published works, please review and evaluate these publications to determine whether they are relevant and should be cited. There is no requirement to cite these works unless the editor has indicated otherwise.

[Note: HTML markup is below. Please do not edit.]

Reviewers' comments: (please note Reviewer #3 is Academic Editor Zypher Jude G. Regencia)

Reviewer's Responses to Questions

**Comments to the Author**

1. If the authors have adequately addressed your comments raised in a previous round of review and you feel that this manuscript is now acceptable for publication, you may indicate that here to bypass the “Comments to the Author” section, enter your conflict of interest statement in the “Confidential to Editor” section, and submit your "Accept" recommendation.

Reviewer #3: (No Response)

**********

2. Is the manuscript technically sound, and do the data support the conclusions?

The manuscript must describe a technically sound piece of scientific research with data that supports the conclusions. Experiments must have been conducted rigorously, with appropriate controls, replication, and sample sizes. The conclusions must be drawn appropriately based on the data presented.

Reviewer #3: Partly

**********

3. Has the statistical analysis been performed appropriately and rigorously?

Reviewer #3: Yes

**********

4. Have the authors made all data underlying the findings in their manuscript fully available?

The PLOS Data policy requires authors to make all data underlying the findings described in their manuscript fully available without restriction, with rare exception (please refer to the Data Availability Statement in the manuscript PDF file). The data should be provided as part of the manuscript or its supporting information, or deposited to a public repository. For example, in addition to summary statistics, the data points behind means, medians and variance measures should be available. If there are restrictions on publicly sharing data—e.g. participant privacy or use of data from a third party—those must be specified.

Reviewer #3: No

**********

5. Is the manuscript presented in an intelligible fashion and written in standard English?

PLOS ONE does not copyedit accepted manuscripts, so the language in submitted articles must be clear, correct, and unambiguous. Any typographical or grammatical errors should be corrected at revision, so please note any specific errors here.

Reviewer #3: Yes

**********

6. Review Comments to the Author

Please use the space provided to explain your answers to the questions above. You may also include additional comments for the author, including concerns about dual publication, research ethics, or publication ethics. (Please upload your review as an attachment if it exceeds 20,000 characters)

Reviewer #3: 1) Title / Framing

The title is clear and matches the analytic approach (LPA) and target group (elementary students).

Small wording tweak: consider “Profiles” rather than “Subtypes” if you want alignment with common LPA language (either is acceptable, but “profiles” is often more standard in this literature).

2) Abstract

Really strong: the sample size and sampling coverage are impressive and give confidence in precision and generalizability within China.

Major concern: the abstract appears to repeat identical odds ratios (e.g., OR = 2.75 and OR = 1.41) across multiple predictors (gender, only-child status, urban/rural, left-behind). That pattern is statistically unlikely and reads like a copy/paste or transcription error. Please verify every OR against Table 4 and correct the abstract accordingly.

Consider adding 1–2 fit statistics (e.g., entropy, rationale for choosing 4-class solution) in a very compact way—PLOS ONE readers appreciate “how you decided.”

3) Introduction / Rationale

Nice job grounding SEC in multiple frameworks (CASEL, OECD, China-localized model) and justifying why CASEL is used here.

The “why person-centered” argument is convincing, especially your example that similar total scores can mask different patterns across domains.

One wording caution: you sometimes imply “developmental profiles,” but the design is cross-sectional. I’d recommend consistently saying “latent profiles in this sample” rather than developmental trajectories (unless you add longitudinal data).

4) Methods

Sampling / Participants

The stratified PPS approach and urban:rural balancing is a real strength; the sampling description is detailed enough for reproducibility.

It would help to clarify the unit of inference: students are nested in classes/schools—did you account for clustering anywhere (e.g., complex survey design in Mplus, robust SEs)? If not, that could inflate significance given N=11k.

Measures

Using the 40-item SEC scale with reported reliabilities and CFA fit indices is good practice. S

I’d like one extra sentence on translation/adaptation (if applicable): Was the instrument administered in Chinese? Was there back-translation or pilot testing?

Procedure / Ethics

The consent/assent process is described clearly, including an opt-out option during administration; that’s reassuring given minors.

Data Availability

There is an internal inconsistency in the rationale for restrictions:

One place says data can’t be shared due to the “ongoing nature” of the project.

Another place frames it as “ethical restrictions protecting the privacy of minors,” with access via the ethics committee / agreements.

These should be unified into one coherent, policy-compliant statement (especially for PLOS ONE).

5) Analytic Strategy (LPA + Predictors)

The LPA selection logic (AIC/BIC/aBIC, entropy, LMR/BLRT) is standard and well explained.

You reject the 5-class model partly because the smallest class is 4.5% (<5%). That’s a defensible heuristic, but I’d recommend phrasing it less as a hard threshold and more as: “small class size raised concerns about stability/interpretability.” Then note whether the 5-class solution was substantively meaningful or just a split of an existing class.

For predictors, you mention R3STEP and multinomial logistic regression. Please ensure the manuscript clearly states:

which profile is the reference class,

coding of predictors (some are dichotomized; grade is collapsed to Grade 3 vs Grades 4–6 in one spot—why not model grade as categorical?), whether you adjusted for clustering.

6) Results

The four-profile solution is intuitive (low → high), and the class proportions are clearly reported.

However, because the profiles are essentially “severity levels” across all five domains, readers may wonder: did you test for more qualitatively distinct patterns (e.g., “socially strong but self-management weak”)? If your indicators are highly correlated, LPA often yields “level” classes. That’s still publishable, but you should acknowledge this and explain the practical value (screening / tiered interventions).

Reporting: Table 3 appears to have at least one formatting/labeling issue (e.g., one row seems truncated around relationship skills). Please check all tables for copyediting artifacts from the submission system.

Biggest statistical red flag remains the repeated ORs as written in the abstract; please correct to match the actual regression table values.

7) Discussion / Conclusion

The discussion on gender and grade patterns is extensive and cites plausible mechanisms (socialization, schooling/SEL exposure, peer context).

Please tighten causal language: with cross-sectional data, statements like “SEL programs lead to…” should be framed as “may contribute” unless you directly measured program exposure or used causal designs.

I’d strongly encourage one short “Implications” subsection translating profiles into actions:

what schools should do differently for low vs low-to-medium vs medium-to-high,

whether cutoffs can be used for screening,

which domains are most actionable.

Overall evaluation (recommendation-style)

This is a large, well-sampled, timely application of person-centered modeling to elementary students’ social-emotional competence in China, and the manuscript is generally well structured and easy to follow. The primary issues that need attention before the paper is publication-ready are (a) correcting/validating the odds ratios reported in the abstract (the repeated OR values look erroneous), (b) resolving the data availability rationale inconsistency, and (c) clarifying a few modeling/reporting choices (clustering, grade coding, and table formatting). Addressing these would substantially strengthen credibility and alignment with PLOS ONE transparency expectations.

**********

7. PLOS authors have the option to publish the peer review history of their article (what does this mean?). If published, this will include your full peer review and any attached files.

If you choose “no”, your identity will remain anonymous but your review may still be made public.

**Do you want your identity to be public for this peer review?** For information about this choice, including consent withdrawal, please see our Privacy Policy.

Reviewer #3: No

**********

[NOTE: If reviewer comments were submitted as an attachment file, they will be attached to this email and accessible via the submission site. Please log into your account, locate the manuscript record, and check for the action link "View Attachments". If this link does not appear, there are no attachment files.]

To ensure your figures meet our technical requirements, please review our figure guidelines: https://journals.plos.org/plosone/s/figures

You may also use PLOS’s free figure tool, NAAS, to help you prepare publication quality figures: https://journals.plos.org/plosone/s/figures#loc-tools-for-figure-preparation.

NAAS will assess whether your figures meet our technical requirements by comparing each figure against our figure specifications.

# Author comment: Author response to Decision Letter 2

**DOI:** 10.1371/journal.pone.0350092.r004

21 Mar 2026

Thank you very much for your comments on this paper. Your comments have important guidance for the improvement of this paper and our scientific research work. These pertinent comments are a great encouragement and support to us. After careful consideration and discussion, we make the following answers according to the review comments. We hope our replies and revision to the manuscript would satisfy the requirements from you. Please feel free to let me know if you have further questions on our revised manuscript. Thank you very much for your consideration and best wishes to you.

We look forward to hearing from you at your earliest convenience.

Attachment Submitted filename: Response_to_Reviewers_auresp_2.docx

# Editor report: Decision Letter 2

**DOI:** 10.1371/journal.pone.0350092.r005

**Authors:** Zypher Jude Regencia

30 Mar 2026

PONE-D-25-19637R2

Profiles of Social-Emotional Competence in Elementary Students: A Latent Profile Analysis

PLOS One

Dear Dr. Wang,

Thank you for submitting your manuscript to PLOS ONE. After careful consideration, we feel that it has merit but does not fully meet PLOS ONE’s publication criteria as it currently stands. Therefore, we invite you to submit a revised version of the manuscript that addresses the points raised during the review process.

I have reviewed the revised manuscript and response letter. The authors have made substantial improvements and have addressed the majority of the reviewer comments. However, a few minor issues remain unresolved. Terminology is still inconsistent, as “profiles” is used in the title while “subtypes” remains in key parts of the manuscript. In addition, Table 3 still contains a visible formatting or typographical artifact that should be corrected. Finally, although the Discussion has been improved, the language should undergo one final review to ensure that no causal implications are made from cross-sectional findings. These are minor issues, and I would support the manuscript after a minor revision.

Please submit your revised manuscript by May 14 2026 11:59PM. If you will need more time than this to complete your revisions, please reply to this message or contact the journal office at plosone@plos.org. When you're ready to submit your revision, log on to https://www.editorialmanager.com/pone/ and select the 'Submissions Needing Revision' folder to locate your manuscript file.

Please include the following items when submitting your revised manuscript:

- A letter that responds to each point raised by the academic editor and reviewer(s). You should upload this letter as a separate file labeled 'Response to Reviewers'.
- A marked-up copy of your manuscript that highlights changes made to the original version. You should upload this as a separate file labeled 'Revised Manuscript with Track Changes'.
- An unmarked version of your revised paper without tracked changes. You should upload this as a separate file labeled 'Manuscript'.

If you would like to make changes to your financial disclosure, please include your updated statement in your cover letter. Guidelines for resubmitting your figure files are available below the reviewer comments at the end of this letter.

If applicable, we recommend that you deposit your laboratory protocols in protocols.io to enhance the reproducibility of your results. Protocols.io assigns your protocol its own identifier (DOI) so that it can be cited independently in the future. For instructions see: https://journals.plos.org/plosone/s/submission-guidelines#loc-laboratory-protocols. Additionally, PLOS ONE offers an option for publishing peer-reviewed Lab Protocol articles, which describe protocols hosted on protocols.io. Read more information on sharing protocols at https://plos.org/protocols?utm_medium=editorial-email&utm_source=authorletters&utm_campaign=protocols.

As the corresponding author, your ORCID iD is verified in the submission system and will appear in the published article. PLOS supports the use of ORCID, and we encourage all coauthors to register for an ORCID iD and use it as well. Please encourage your coauthors to verify their ORCID iD within the submission system before final acceptance, as unverified ORCID iDs will not appear in the published article. *Only* the individual author can complete the verification step; PLOS staff *cannot* verify ORCID iDs on behalf of authors.

We look forward to receiving your revised manuscript.

Kind regards,

Zypher Jude G. Regencia, Ph.D.

Academic Editor

PLOS One

Journal Requirements:

1. If the reviewer comments include a recommendation to cite specific previously published works, please review and evaluate these publications to determine whether they are relevant and should be cited. There is no requirement to cite these works unless the editor has indicated otherwise.

2. Please review your reference list to ensure that it is complete and correct. If you have cited papers that have been retracted, please include the rationale for doing so in the manuscript text, or remove these references and replace them with relevant current references. Any changes to the reference list should be mentioned in the rebuttal letter that accompanies your revised manuscript. If you need to cite a retracted article, indicate the article’s retracted status in the References list and also include a citation and full reference for the retraction notice.

**********

[NOTE: If reviewer comments were submitted as an attachment file, they will be attached to this email and accessible via the submission site. Please log into your account, locate the manuscript record, and check for the action link "View Attachments". If this link does not appear, there are no attachment files.]

To ensure your figures meet our technical requirements, please review our figure guidelines: https://journals.plos.org/plosone/s/figures

You may also use PLOS’s free figure tool, NAAS, to help you prepare publication quality figures: https://journals.plos.org/plosone/s/figures#loc-tools-for-figure-preparation.

NAAS will assess whether your figures meet our technical requirements by comparing each figure against our figure specifications.

# Author comment: Author response to Decision Letter 3

**DOI:** 10.1371/journal.pone.0350092.r006

4 Apr 2026

Thank you very much for your comments on this paper. Your comments have important guidance for the improvement of this paper and our scientific research work. These pertinent comments are a great encouragement and support to us. After careful consideration and discussion, we make the following answers according to the review comments. We hope our replies and revision to the manuscript would satisfy the requirements from you. Please feel free to let me know if you have further questions on our revised manuscript. Thank you very much for your consideration and best wishes to you.

We look forward to hearing from you at your earliest convenience.

Attachment Submitted filename: Response to Reviewer.docx

# Editor report: Decision Letter 3

**DOI:** 10.1371/journal.pone.0350092.r007

**Authors:** Zypher Jude Regencia

27 Apr 2026

PONE-D-25-19637R3

Profiles of Social-Emotional Competence in Elementary Students: A Latent Profile Analysis

PLOS One

Dear Dr. Wang,

Thank you for submitting your manuscript to PLOS ONE. After careful consideration, we feel that it has merit but does not fully meet PLOS ONE’s publication criteria as it currently stands. Therefore, we invite you to submit a revised version of the manuscript that addresses the points raised during the review process.

I have reviewed the current version as the editor and most of the major revisions appear to have been addressed. The manuscript has improved, particularly in the clarification of the latent profile approach, the justification for the four profile model, the description of the translation and adaptation of the instrument, the handling of clustering, and the addition of practical implications.

Before final submission, however, I suggest doing one more careful technical cleanup to avoid issues during editorial or reviewer checking.

First, please ensure that the terminology is consistent throughout the manuscript. The response indicates that “subtypes” was changed to “profiles,” but some instances of “subtype” still appear in the cover letter and manuscript. Please replace these with “profile” or “profile membership” as appropriate.

Second, please recheck the odds ratios in the abstract and Table 4 against the original Mplus output. Several OR values remain very close to one another, such as 2.754, 2.752, 2.746, and 2.752. If these are correct, the response to the reviewer should explicitly state that the values were verified against the original output. If not, please correct them accordingly.

Third, please correct the typographical issue in Table 3. The Social awareness row appears to show “3.04 ± 016” and should likely be “3.04 ± 0.16.”

Fourth, please soften any remaining causal language, since the study is cross sectional. Phrases such as “impact of demographic variables,” “effects of these demographic variables,” and “significantly influenced SEC” may be revised to “were associated with SEC profiles,” “were linked to profile membership,” or “students with these characteristics were more likely to belong to specific profiles.”

Fifth, please standardize the Data Availability Statement across the manuscript, cover letter, and submission form. Since the data involve minors, the statement should clearly identify the ethics committee, the valid contact information, the criteria for data access, and the requirement for a confidentiality or data use agreement. Please also check the email formatting in the cover letter, as there appears to be an extra space in the ethics committee email address.

Finally, in the Data Analysis section, the sentence referring to demographic variables “such as gender and age” should be revised because age was not the actual predictor reported. Please list the actual variables used, including gender, grade, only child status, school location, and left behind status.

Overall, the manuscript is much improved and the revisions are generally responsive to the comments. I recommend addressing the above cleanup points before resubmission so that the revised version is internally consistent and reviewer ready.

Please submit your revised manuscript by Jun 11 2026 11:59PM. If you will need more time than this to complete your revisions, please reply to this message or contact the journal office at plosone@plos.org. When you're ready to submit your revision, log on to https://www.editorialmanager.com/pone/ and select the 'Submissions Needing Revision' folder to locate your manuscript file.

Please include the following items when submitting your revised manuscript:

- A letter that responds to each point raised by the academic editor and reviewer(s). You should upload this letter as a separate file labeled 'Response to Reviewers'.
- A marked-up copy of your manuscript that highlights changes made to the original version. You should upload this as a separate file labeled 'Revised Manuscript with Track Changes'.
- An unmarked version of your revised paper without tracked changes. You should upload this as a separate file labeled 'Manuscript'.

If you would like to make changes to your financial disclosure, please include your updated statement in your cover letter. Guidelines for resubmitting your figure files are available below the reviewer comments at the end of this letter.

If applicable, we recommend that you deposit your laboratory protocols in protocols.io to enhance the reproducibility of your results. Protocols.io assigns your protocol its own identifier (DOI) so that it can be cited independently in the future. For instructions see: https://journals.plos.org/plosone/s/submission-guidelines#loc-laboratory-protocols. Additionally, PLOS ONE offers an option for publishing peer-reviewed Lab Protocol articles, which describe protocols hosted on protocols.io. Read more information on sharing protocols at https://plos.org/protocols?utm_medium=editorial-email&utm_source=authorletters&utm_campaign=protocols.

As the corresponding author, your ORCID iD is verified in the submission system and will appear in the published article. PLOS supports the use of ORCID, and we encourage all coauthors to register for an ORCID iD and use it as well. Please encourage your coauthors to verify their ORCID iD within the submission system before final acceptance, as unverified ORCID iDs will not appear in the published article. *Only* the individual author can complete the verification step; PLOS staff *cannot* verify ORCID iDs on behalf of authors.

We look forward to receiving your revised manuscript.

Kind regards,

Zypher Jude G. Regencia, Ph.D.

Academic Editor

PLOS One

Journal Requirements:

1. If the reviewer comments include a recommendation to cite specific previously published works, please review and evaluate these publications to determine whether they are relevant and should be cited. There is no requirement to cite these works unless the editor has indicated otherwise.

2. Please review your reference list to ensure that it is complete and correct. If you have cited papers that have been retracted, please include the rationale for doing so in the manuscript text, or remove these references and replace them with relevant current references. Any changes to the reference list should be mentioned in the rebuttal letter that accompanies your revised manuscript. If you need to cite a retracted article, indicate the article’s retracted status in the References list and also include a citation and full reference for the retraction notice.

[NOTE: If reviewer comments were submitted as an attachment file, they will be attached to this email and accessible via the submission site. Please log into your account, locate the manuscript record, and check for the action link "View Attachments". If this link does not appear, there are no attachment files.]

To ensure your figures meet our technical requirements, please review our figure guidelines: https://journals.plos.org/plosone/s/figures

You may also use PLOS’s free figure tool, NAAS, to help you prepare publication quality figures: https://journals.plos.org/plosone/s/figures#loc-tools-for-figure-preparation.

NAAS will assess whether your figures meet our technical requirements by comparing each figure against our figure specifications.

# Author comment: Author response to Decision Letter 4

**DOI:** 10.1371/journal.pone.0350092.r008

28 Apr 2026

Thank you very much for your comments on this paper. Your comments have important guidance for the improvement of this paper and our scientific research work. These pertinent comments are a great encouragement and support to us. After careful consideration and discussion, we make the following answers according to the review comments. We hope our replies and revision to the manuscript would satisfy the requirements from you. Please feel free to let me know if you have further questions on our revised manuscript. Thank you very much for your consideration and best wishes to you.

We look forward to hearing from you at your earliest convenience.

Attachment Submitted filename: Response to Reviewers.pdf

# Editor report: Decision Letter 4

**DOI:** 10.1371/journal.pone.0350092.r009

**Authors:** Zypher Jude Regencia

3 May 2026

PONE-D-25-19637R4

Profiles of Social-Emotional Competence in Elementary Students: A Latent Profile Analysis

PLOS One

Dear Dr. Wang

Thank you for submitting your manuscript to PLOS ONE. After careful consideration, we feel that it has merit but does not fully meet PLOS ONE’s publication criteria as it currently stands. Therefore, we invite you to submit a revised version of the manuscript that addresses the points raised during the review process.

I have reviewed the revised files and note that most of the previous concerns have been addressed satisfactorily. However, a few minor revisions are still needed before the manuscript can proceed to the next stage.

First, please carefully recheck the reported odds ratios in the Abstract and Table 4. Some values reported in the Abstract as odds ratios appear to correspond to the regression coefficients in Table 4 rather than the OR values. Please verify these against the original Mplus output and ensure that the Abstract, Table 4, and response to reviewers are fully consistent.

Second, please further soften the remaining causal language in the manuscript, given the cross-sectional design. Phrases suggesting influence, effects, or mechanisms should be revised to associative wording, such as “were linked to profile membership,” “were more likely to belong to specific profiles,” or “were associated with SEC profiles.”

Third, please ensure that all tracked changes are accepted in the clean version of the manuscript, unless a tracked version is specifically required. This will help avoid the appearance that previously revised terms, such as “subtype,” remain in the manuscript.

Finally, please confirm that the Data Availability Statement is identical across the manuscript, cover letter, and submission form, including the ethics committee name, contact email, access criteria, and the requirement for a data confidentiality agreement.

These are minor but important editorial points. I would be pleased to consider the manuscript further once these revisions have been completed.

Please submit your revised manuscript by Jun 17 2026 11:59PM. If you will need more time than this to complete your revisions, please reply to this message or contact the journal office at plosone@plos.org. When you're ready to submit your revision, log on to https://www.editorialmanager.com/pone/ and select the 'Submissions Needing Revision' folder to locate your manuscript file.

Please include the following items when submitting your revised manuscript:

- A letter that responds to each point raised by the academic editor and reviewer(s). You should upload this letter as a separate file labeled 'Response to Reviewers'.
- A marked-up copy of your manuscript that highlights changes made to the original version. You should upload this as a separate file labeled 'Revised Manuscript with Track Changes'.
- An unmarked version of your revised paper without tracked changes. You should upload this as a separate file labeled 'Manuscript'.

If you would like to make changes to your financial disclosure, please include your updated statement in your cover letter. Guidelines for resubmitting your figure files are available below the reviewer comments at the end of this letter.

If applicable, we recommend that you deposit your laboratory protocols in protocols.io to enhance the reproducibility of your results. Protocols.io assigns your protocol its own identifier (DOI) so that it can be cited independently in the future. For instructions see: https://journals.plos.org/plosone/s/submission-guidelines#loc-laboratory-protocols. Additionally, PLOS ONE offers an option for publishing peer-reviewed Lab Protocol articles, which describe protocols hosted on protocols.io. Read more information on sharing protocols at https://plos.org/protocols?utm_medium=editorial-email&utm_source=authorletters&utm_campaign=protocols.

As the corresponding author, your ORCID iD is verified in the submission system and will appear in the published article. PLOS supports the use of ORCID, and we encourage all coauthors to register for an ORCID iD and use it as well. Please encourage your coauthors to verify their ORCID iD within the submission system before final acceptance, as unverified ORCID iDs will not appear in the published article. *Only* the individual author can complete the verification step; PLOS staff *cannot* verify ORCID iDs on behalf of authors.

We look forward to receiving your revised manuscript.

Kind regards,

Zypher Jude G. Regencia, Ph.D.

Academic Editor

PLOS One

Journal Requirements:

If the reviewer comments include a recommendation to cite specific previously published works, please review and evaluate these publications to determine whether they are relevant and should be cited. There is no requirement to cite these works unless the editor has indicated otherwise.

Please review your reference list to ensure that it is complete and correct. If you have cited papers that have been retracted, please include the rationale for doing so in the manuscript text, or remove these references and replace them with relevant current references. Any changes to the reference list should be mentioned in the rebuttal letter that accompanies your revised manuscript. If you need to cite a retracted article, indicate the article’s retracted status in the References list and also include a citation and full reference for the retraction notice.

To ensure your figures meet our technical requirements, please review our figure guidelines: https://journals.plos.org/plosone/s/figures

You may also use PLOS’s free figure tool, NAAS, to help you prepare publication quality figures: https://journals.plos.org/plosone/s/figures#loc-tools-for-figure-preparation.

NAAS will assess whether your figures meet our technical requirements by comparing each figure against our figure specifications.

# Author comment: Author response to Decision Letter 5

**DOI:** 10.1371/journal.pone.0350092.r010

4 May 2026

Thank you very much for your comments on this paper. Your comments have important guidance for the improvement of this paper and our scientific research work. These pertinent comments are a great encouragement and support to us. After careful consideration and discussion, we make the following answers according to the review comments. We hope our replies and revision to the manuscript would satisfy the requirements from you. Please feel free to let me know if you have further questions on our revised manuscript. Thank you very much for your consideration and best wishes to you.

We look forward to hearing from you at your earliest convenience.

Attachment Submitted filename: Response_to_Reviewers_auresp_5.docx

# Editor report: Decision Letter 5

**DOI:** 10.1371/journal.pone.0350092.r011

**Authors:** Zypher Jude Regencia

11 May 2026

Profiles of Social-Emotional Competence in Elementary Students: A Latent Profile Analysis

PONE-D-25-19637R5

Dear Dr. Wang,

We’re pleased to inform you that your manuscript has been judged scientifically suitable for publication and will be formally accepted for publication once it meets all outstanding technical requirements.

I only have a few minor comments that may be addressed during the final editorial or production stage:

First, please check the statement in the Abstract regarding non-left-behind children. The odds ratio of 4.094 appears to correspond to the medium to high level group in Table 4, not the low to medium level group. Please revise the wording accordingly to ensure consistency between the Abstract and the results table.

Second, although the manuscript has been substantially improved, some remaining phrases may still imply causality, such as “predictive effects,” “influences,” “mechanisms,” “enhance,” “impact,” and similar wording. Since the study uses a cross-sectional design, please ensure that these are softened where appropriate and framed in terms of associations rather than causal relationships.

Lastly, please make sure that the final clean version does not contain any residual tracked-change text or merged wording from the revision process.

Within one week, you’ll receive an e-mail detailing the required amendments. When these have been addressed, you’ll receive a formal acceptance letter and your manuscript will be scheduled for publication.

An invoice will be generated when your article is formally accepted. Please note, if your institution has a publishing partnership with PLOS and your article meets the relevant criteria, all or part of your publication costs will be covered. Please make sure your user information is up-to-date by logging into Editorial Manager at Editorial Manager® and clicking the ‘Update My Information' link at the top of the page. For questions related to billing, please contact billing support.

If your institution or institutions have a press office, please notify them about your upcoming paper to help maximize its impact. If they’ll be preparing press materials, please inform our press team as soon as possible -- no later than 48 hours after receiving the formal acceptance. Your manuscript will remain under strict press embargo until 2 pm Eastern Time on the date of publication. For more information, please contact onepress@plos.org.

Kind regards,

Zypher Jude G. Regencia, Ph.D.

Academic Editor

PLOS One

Additional Editor Comments (optional):

Reviewers' comments:

# Editor report: Acceptance letter

**DOI:** 10.1371/journal.pone.0350092.r012

**Authors:** Zypher Jude Regencia

PONE-D-25-19637R5

PLOS One

Dear Dr. Wang,

I'm pleased to inform you that your manuscript has been deemed suitable for publication in PLOS One. Congratulations! Your manuscript is now being handed over to our production team.

At this stage, our production department will prepare your paper for publication. This includes ensuring the following:

* All references, tables, and figures are properly cited

* All relevant supporting information is included in the manuscript submission,

* There are no issues that prevent the paper from being properly typeset

You will receive further instructions from the production team, including instructions on how to review your proof when it is ready. Please keep in mind that we are working through a large volume of accepted articles, so please give us a few days to review your paper and let you know the next and final steps.

Lastly, if your institution or institutions have a press office, please let them know about your upcoming paper now to help maximize its impact. If they'll be preparing press materials, please inform our press team within the next 48 hours. Your manuscript will remain under strict press embargo until 2 pm Eastern Time on the date of publication. For more information, please contact onepress@plos.org.

You will receive an invoice from PLOS for your publication fee after your manuscript has reached the completed accept phase. If you receive an email requesting payment before acceptance or for any other service, this may be a phishing scheme. Learn how to identify phishing emails and protect your accounts at https://explore.plos.org/phishing.

If we can help with anything else, please email us at customercare@plos.org.

Thank you for submitting your work to PLOS ONE and supporting open access.

Kind regards,

PLOS ONE Editorial Office Staff

on behalf of

Dr. Zypher Jude G. Regencia

Academic Editor

PLOS One
